# Zoonoses in dog and cat shelters in North-East Italy: update on emerging, neglected and known zoonotic agents

**DOI:** 10.3389/fvets.2024.1490649

**Published:** 2024-11-27

**Authors:** Elisa Mazzotta, Laura Lucchese, Michela Corrò, Letizia Ceglie, Patrizia Danesi, Katia Capello, Alda Natale

**Affiliations:** Istituto Zooprofilattico Sperimentale delle Venezie, Legnaro, Italy

**Keywords:** emergent zoonoses, neglected zoonoses, dog shelter, cat shelter, One Health

## Abstract

**Introduction:**

Shelters for stray dogs and cats deserve careful monitoring for zoonotic risk, as they represent a crucial point for prevention and control of infection spread. Data sorting to prioritize zoonotic agents in a geographic area need constant updating, but no regular official programs are ongoing, to allow an efficient risk survey for these animal species. This study aimed to conduct a comprehensive investigation of the prevalence of certain known, potential and emerging zoonoses within the framework of the routine monitoring of dog and cat shelters in North-East Italy.

**Methods:**

A total of 389 cats and 257 dogs housed in public veterinary services shelters and feline colonies were included in the present investigation. The animals originated from the provinces of Padua, Venice, Rovigo, Vicenza, Verona, Trento and Bolzano. Serological, molecular and microbiological diagnostics were implemented to investigate the prevalence of *Leptospira* sp., *Brucella canis, Leishmania infantum*, dermatophytes, gastrointestinal parasites, antimicrobial-resistant bacteria, *Capnocytophaga* sp., *Bartonella* sp., Norovirus, Rotavirus A, Cowpox virus, Mammalian Orthoreovirus, Hepatitis E virus, SARS-CoV-2 and Influenza A virus.

**Results:**

Data about some known zoonoses (e.g., serological positivity of *Leishmania infantum* 25% and *Leptospira* sp. 44.3% in dogs, and *Bartonella henselae* 70% in cats) resulted aligned with previous research and recent reports, whereas there was a notable occurrence of some potential, emerging and neglected pathogens (e.g., Mammalian Orthoreovirus 0.38% in dogs and 2.83% in cats). For some other agents (e.g., dermatophytes in dogs and in cats) the prevalence resulted lower than expected.

**Discussion:**

The prevention of the zoonotic risk requires a re-examination of the complex interaction between humans, animals, and environment. This is of particular importance in settings like companion animal shelters, which serve as key sites for disease monitoring and zoonotic risk mitigation. The study highlights the need to monitor and prioritize the zoonotic pathogens, to implement and constantly update surveillance and specific training programs for the kennels’ operators, and management of epidemiological risks.

## 1 Introduction

Dogs and cats have coexisted with humans for thousands of years, and as domesticated animals, they provide significant psychological benefits for our contemporary, city-centered society. In addition, there are recognized links between companion animals and human infections caused by various zoonotic agents, including bacteria, fungi, viruses and parasites (1). Whilst certain pathogens may be widely recognized, others are newly emerging, under-researched or have unexplored potential to spread between animals and humans, yet are not currently subject to extensive reporting or scientific analysis. A number of zoonoses affecting pets are well known in Italy [e.g., leptospirosis (2–4), leishmaniosis (5–9), dermatophytosis (10), echinococcosis (11, 12), bartonellosis (13–15), gastrointestinal parasites and vector-borne diseases (16, 17)], however the lack of official surveillance programs results in a scarcity of information about the prevalence of these diseases and pathogens. There are also emerging or rare infections for which the prevalence is completely unknown. For example, *Capnocytophaga canimorsus*, commonly part of the oral bacterial microbiota of dogs and cats, is reported to cause fatal infections in humans (18–21). Furthermore, the zoonotic role of antibiotic-resistant bacteria (22–25) or other pathogens such as *Brucella canis* are still poorly investigated (26–28). Hepatitis E virus (HEV) detected in both domestic and wild species (e.g., swine, wild boar, dog, cat, deer, rabbit, mongoose) (29–39), and Cowpox virus recently identified (40–42), are pathogens agents with unknown prevalence among pets in Italy. In the context of the recent SARS-CoV-2 pandemic, both experimental evidence and observations of natural infections have demonstrated the susceptibility of companion animals (43–49). In addition, emerging and potential zoonotic pathogens responsible for respiratory or gastrointestinal syndromes in humans, including Influenza A viruses (50, 51), Rotaviruses (1, 52, 53), Mammalian Orthoreovirus (54) and Noroviruses (55, 56), need to be further investigated. For these reasons, the lack of official reporting of emerging and neglected zoonoses calls attention to the need to improve surveillance and promptly identify potential outbreaks and spillovers. These animals can serve as environmental sentinels or reservoirs for numerous potential zoonotic diseases, either through direct or indirect transmission pathways, which are often overlooked (57–59). As previously reported, fatal epizootic and/or potentially epidemic infectious diseases such as respiratory, neurological and systemic streptococcal infections (in dogs and cats), canine influenza, haemorrhagic respiratory *Escherichia coli* infection (in dogs) and virulent systemic feline Calicivirus infection, have all emerged within animal shelter populations over the past decade (60–62).

Recent official data about the population of stray dogs and cats in North-East Italy (Veneto region) reported a number of 2,706 accesses in dogs’ sanitary shelters, 790 accesses in kennel shelters. Among these dogs, 1,279 dogs were returned to the owner, and 867 were adopted. The data reported that in Veneto the Veterinary Services neutered 8,842 cats as part of the stray prevention campaign (catch and release).^1^ Despite this scenario, for companion animals, there are no organized infectious and zoonotic diseases surveillance plans according to updated public veterinary services. Few studies have reported specific surveys of shelter demographics, infection control practices and policies, as well as awareness and concern about infectious and zoonotic diseases (63–65). The day-to-day management of the facilities primarily falls upon non-specialized personnel. The facilities pose a significant health and hygiene risk for both animals and humans due to the high turnover of animals, varying in ages, breeds, and origins, confined in a small space. Additionally, the frequent presence of volunteer staff, often inadequately trained, exacerbates these risks. Staff may expose themselves directly to health risks, and/or unintentionally spreading infections.

The aim of the present study was to investigate the prevalence and the circulation of different known, emergent, neglected or potential zoonotic pathogens among cats and dogs sheltered, in order to update the information on these zoonotic agents and suggest a prioritization for surveillance programs should be implement. In addition, this study reports the implementation of protocols for screening and prevention of zoonotic diseases, in order to improve the management for health protection in a One Health perspective.

## 2 Materials and methods

### 2.1 Sampling and reference populations

Dog and cat shelters of Veneto and Trentino-Alto Adige regions were the reference populations used to investigate the circulation of the selected zoonotic agents, as reported below.

Since we could not find similar studies to estimate the prevalence of the zoonotic agents under evaluation, we chose to use the prevalence level that yields the maximum sample size. This approach corresponds to a prevalence of 50% (with a 95% confidence interval and a desired absolute precision of 5%). Given the objective to estimate the prevalence at animal level, we adopted a simple random sampling approach to select the animals from the reference population (66). An initial sample size of 385 animals per group was considered. All animals were randomly selected among the newly introduced at the shelter at the time of collecting samples. Animal data including neutered/intact sex, age, breed, clinical signs, vaccination status, and origin were recorded when available. Blood, urine, feces, rectal (R) and oropharyngeal (OP) swabs were collected by veterinarians for diagnostic, therapeutic or prophylactic purposes and the panel of research analyses was proposed as additional investigation.

Blood was collected in K3EDTA tubes and urine collected in sterile urine tubes. Specific Ellinghausen–McCullough–Johnson–Harris medium (EMJH) was used to preserve urine (*n* = 144 dogs) for *Leptospira* isolation.

### 2.2 Molecular pre-analytical steps

Nucleic acid extraction from dog urine and K3EDTA blood samples addressed to *Leptospira* spp. investigation was performed on the KingFisher™ Flex Purification System (Thermo Fisher Scientific Inc.) platform using the ID Gene® Mag Universal Extraction Kit (IDVet, Grabels, France), following the manufacturer’s instructions. The same kit was used for the extraction of nucleic acids from OP and rectal swabs and fecal samples, after the addition of PK (Qiagen GmbH, Germany) and an incubation at 70°C for 10′. The High Pure PCR Template Preparation Kit (Roche Diagnostics Mannheim, Germany) was used to extract *Bartonella henselae* and/or *B. clarridgeiae* DNA.

After a pretreatment step, in order to reduce the possible inhibition factors and increase the sensitivity of the assay, the viral RNA addressed to the search of Hepeviruses was extracted from 200 μL of stool supernatants, using the ID Gene™ Mag Universal extraction Kit (IDVet Genetics, Grabels, France) on the above-mentioned instrument. Briefly, fecal material thawed at 4°C was diluted 1:5 in PBS (pH 7.4), shaken vigorously and incubated overnight at 4°C. The day after, stool samples were centrifuged at 16000×*g* for 5 min to recover viral suspensions in the supernatants.

A universal heterologous RNA internal control was included to validate the negative results obtained in molecular analyses to detect Orthohepevirus A and C, Rotavirus A, Influenza A, and SARS-CoV-2 RNA. In detail, the Intype IC-RNA (Indical Bioscience GmbH, Leipzig, Germany) was added to each sample during the extraction step at a ratio of 1:10 of the total elution volume. The primers EGFP 1- F and EGFP2-R and the EGFP-HEX probe according to Hoffmann et al. (67) were then used for co-amplification. All PCR reactions were carried out on the CFX 1000 (Bio-Rad) platform or on the QuantStudio 5 (Thermo Fisher) platform and data analyses were performed by means of specific SWs, namely Bio-Rad CFX Maestro 1.1, QuantStudio™ Design & Analysis Software (DESKTOP) VERSION 2.6, respectively. All nucleic acids extraction included a negative control (water) to identify any possible contamination, while all amplification included a positive control for each target considered and a negative master mix control.

Molecular analyses targets and reaction’s conditions are summarized in Supplementary material.

### 2.3 Pathogens investigated

The list of investigated pathogens and biological samples is summarized in Table 1.

**Table 1:** List of zoonotic pathogens acknowledged for health monitoring, categorized by species.

Pathogen	Dog	Cat	Biological samples	Analysis
*Leptospira* spp.	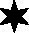		Serum, blood K3EDTA, Urine	Serological, molecular, isolation
*Leishmania infantum*	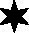		Serum	Serological
*Bartonella henselae/Bartonella clarridgeiae*		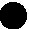	Serum, blood K3EDTA	Serological, molecular
Bacteria ESBL, MRCPS, VRE	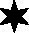	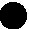	Rectal swab/Feces Oral swab	Microbiological, Bacterial strain typing
*Brucella canis*	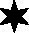		Serum	Serological
Dermatophytes	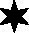	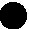	Hair/crust/skin lesions	Microbiological
Nematode and Coccidia	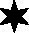	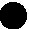	Feces	Flotation
Cestoda	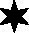	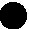	Feces	Flotation
*Capnocytophaga* sp.	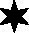	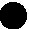	Oral swab	Microbiological, molecular
Coronavirus (SARS-CoV-2)	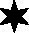	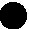	Serum Rectal swab/Feces Oral swab	Serology, molecular
Hepatitis E virus	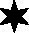	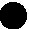	Serum, Feces	Serology, molecular
Norovirus	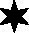	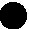	Rectal swab/Feces	Molecular
Rotavirus A	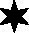	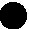	Rectal swab/Feces	Molecular
Cowpox virus		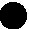	Hair/crust/skin lesions	Molecular
Mammalian Orthoreovirus	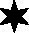	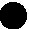	Rectal swab/Feces	Molecular
Influenza A virus	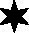	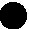	Rectal swab/Feces Oral swab	Molecular

Each pathogen’s biological samples examined and associated laboratory analyses are provided. ESBL, Extended-Spectrum Beta-Lactamases; MRSCP, Methicillin-resistant coagulase positive Staphylococci; VRE, Vancomycin resistant Enterococci.

#### 2.3.1 Leptospira

##### 2.3.1.1 Molecular investigations

*Leptospira* spp. DNA was investigated in blood (*n* = 144) and urine (*n* = 257) of dogs by real-time PCR (rPCR), as reported below.

*Leptospira* spp. DNAs were analyzed by means of a rPCR protocol, targeting a 87-bp genomic fragment within the rrs (16S) gene of pathogenic leptospires, as described in prior studies (2, 68), using the Path-ID™ qPCR Master Mix (Thermo Fisher Scientific Inc.). Positive samples were genotyped at the National Reference Center for Leptospirosis (Istituto Zooprofilattico Sperimentale della Lombardia e dell’Emilia Romagna, IZSLER) using Multi-locus Sequence Typing (MLST) (69).

##### 2.3.1.2 Culture

Leptospira isolation was attempted via culture examination (*n* = 144 dogs) as previously described method (70).

##### 2.3.1.3 Serology

A total of 257 canine sera were submitted for the examination of specific antibodies against *Leptospira* spp. through the microagglutination technique (MAT) (70). The antigen panel included 11 serovars distributed by the Italian Reference Center for Animal Leptospirosis (71).

#### 2.3.2 Leishmania infantum

##### 2.3.2.1 Serology

A total of 257 canine sera were tested for anti-*Leishmania infantum* antibodies by using the indirect immunofluorescence assay (IFAT), with a cut-off of 1:40 (72). According to the WOAH Terrestrial Manual (72), cut-off ranging from 1:40 to 1:80 indicate exposure to the pathogen, while higher titres (≥1:160) typically concur with a confirmed diagnosis of leishmaniosis. The sera were analyzed using doubling dilutions from 1:40 to the final positive titer.

#### 2.3.3 Bartonella

##### 2.3.3.1 Molecular investigations

*Bartonella* spp. DNA was investigated in blood of cats (*n* = 386) by rPCR targeting a 110 bp-fragment belonging to the citrate synthase gene, as previously described (73). Amplification’s products were sequenced in order to identify *Bartonella henselae* and/or *B. clarridgeiae* species.

##### 2.3.3.2 Serology

A total of 389 feline sera were analyzed to detect anti-*Bartonella henselae* antibodies by employing IFAT with a commercial kit (Biopronix, manufactured by Agrolabo in Turin, Italy), based on whole antigen *B. henselae,* using a cut-off of 1:64. The sera were analyzed using doubling dilutions from 1:64 to 1:256. Samples with titers ≥1:256 were not titrated further but were considered strong positive samples.

To assess serological positivity indicative of active infection, higher cut-offs (≥1:128) were considered (74).

#### 2.3.4 Antibiotic-resistant bacteria

##### 2.3.4.1 Culture

A total of 257 canine samples and 389 feline OP and R swabs were collected using sterile swabs. Samples were refrigerated (+4°C) until analysis and the isolated bacterial culture frozen (−80°C). OP swabs were used to isolate methicillin-resistant coagulase-positive *Staphylococci* (MRCPS), R swabs to isolate vancomycin-resistant *Enterococci* (VRE), Extended-Spectrum Beta-Lactamases (ESBL)-producing *Enterobacteria*, and *Pseudomonas* spp. Bacterial isolation was performed by using specific enrichment/selective media to detect the presence of ESBL-producing *Enterobacteria* and *Pseudomonas aeruginosa* (1 mg/L cefotaxime brain heart infusion broth/McConkey Agar +1 mg/L cefotaxime), MRCPS (6.5%NaCL broth/CHROMagar TM MRSA II), VRE (VRE broth/CHROMID agar).

#### 2.3.5 Brucella canis

##### 2.3.5.1 Serology

A total of 257 canine sera were analyzed by a validated reference protocol for detection of anti-*Brucella canis* antibodies (microplate-serum agglutination test (mSAT) National Reference Centre for Brucellosis (CNRB) – IZS Teramo) (75). The sera were analyzed using doubling dilutions ranging from 1:20 to 1:640 and incubated at 37°C for 48 h. If serological titers were 1:20 or higher, the samples were sent to the national reference laboratory (CNRB) and WOAH (World Organization of Animal Health) for brucellosis for confirmatory tests, such as the complement fixation tests (CFTs), immunofluorescence tests (IFs), and bacterial isolation from blood and urine.

#### 2.3.6 Parasites and dermatophytes

##### 2.3.6.1 Copromicroscopic methods

A total of 177 canine and 143 feline stool samples were tested by conventional method consisting of sedimentation followed by sodium nitrate flotation (specific gravity 1.3) (76). Each fecal floatation was observed on a slide under a light microscope for the morphometric evaluation of helminths eggs according to existing keys for intestinal parasites (77).

##### 2.3.6.2 Molecular investigations

Specimens positive to cestode eggs were submitted to a multiplex PCR (CMPCR) for identification of *Echinococcus granulosus (E. granulosus)*, *Echinococcus multilocularis (E. multilocularis)* and Tenidae as previously described by Citterio et al. (78).

##### 2.3.6.3 Mycological culture

A total of 389 cats’ and 257 dogs’ individual hair specimens collected by brushing techniques were cultured on mycobiotic agar for 14 days at 25°C. Fungal colonies were identified phenotypically to genus or species on the basis of colony morphology and microscopic examination, following established methods (79).

All testing procedures and protocols have been standardized and are presently being utilized at the IZSVE laboratories.

#### 2.3.7 Capnocytophaga

##### 2.3.7.1 Culture

*Capnocytophaga* sp. isolation was attempted in 257 dogs and 389 cats’ OP swabs. The isolation method consists of the use of a blood agar medium containing gentamicin, as described by Van Dam et al. (80), and an enrichment broth for microaerophilic microorganisms (Thioglycollate broth – THG) to encourage the growth of the bacterium even under low-burden conditions. Suspected colonies are recognized through their macroscopic and microscopic characteristics as Gram-negative bacilli, as well as through positive reactions to certain biochemical tests such as catalase and oxidase. The microbiology was performed by culture examination utilizing strains of *C. canimorsus* NCTC 12242 and *C. cynodegmi* NCTC 12243 as positive controls, according to the guidelines outlined by Van Dam et al. Species identification was confirmed by Matrix Assisted Laser Desorption/Ionization Time-Of-Flight Mass Spectrometry (MALDI-TOF MS) in accordance with the methodology recommended by the manufacturer of the instrument, the MALDI Biotyper Microflex LT (Bruker Daltonics).

##### 2.3.7.2 Molecular investigation

A total of 257 dogs’ and 389 cats’ OP swabs were screened for *Capnocytophaga* spp., using 2 different rPCR protocols targeting two specific and differentiating fragments included in the *rpoB* gene (80).

#### 2.3.8 SARS-CoV-2

##### 2.3.8.1 Molecular investigation

SARS-CoV-2 RNA detection was attempted in both dogs’ and cats’ (*n* = 257 dogs; *n* = 389 cats) OP and R swabs using the rRT-PCR method, Corman et al. (81).

##### 2.3.8.2 Serology

The serum samples of both dogs (*n* = 257) and cats (*n* = 389) were tested for specific anti-SARS-CoV-2 antibodies via commercial serological tests utilizing ELISA kits (ID Screen® – SARS-CoV-2 Double Antigen Multi-species, Innovative Diagnostics, Grabels, France). In cases of suspected infection, a serum neutralization and plaque reduction test (Plaque Reduction Neutralization Test-PRNT) was performed, as previously reported (47, 82).

#### 2.3.9 Hepatitis E virus

##### 2.3.9.1 Molecular investigation

Orthohepesvirus (A and C), viral RNAs, extracted as previously described, were investigated in rectal swabs (*n* = 257 dogs; *n* = 389 cats) and individual stools (2 g) of 177 dogs and 143 cats by realtime reverse transcription PCR (rRT-PCR). Briefly, Orthohepevirus rRT-PCR protocols were species specific and targeted a 70 bp and a 73 bp fragments within the ORF 3 genomic region, for the Orthohepevirus A (83) and Orthohepevirus C species, respectively (84).

##### 2.3.9.2 Serology

All canine and feline sera were tested for specific anti-HEV antibodies using a commercial multispecies ELISA assay (ID Screen® Hepatitis E Indirect Multi-species, Innovative Diagnostics, Grabels, France).

#### 2.3.10 Norovirus, rotavirus A and mammalian orthoreovirus (MRV)

##### 2.3.10.1 Molecular investigation

Norovirus end point RT-PCR targeted a fragment of 300 bp within the RNA-dependent RNA polymerase (RdRp) that is highly preserved in the Caliciviridae family (85) and was applied to all rectal swabs collected.

Rotavirus genotype A was investigated by implementing a rRT-PCR protocols targeting Nsp3 gene for Rotavirus A (86) on RNAs extracted from the same samples.

Mammalian Orthoreovirus (MRV) detection was attempted in both dogs’ and cats’ samples by implementing an end-point RT-PCR protocol that has already been utilized for swine samples in the laboratory for screening purposes. This technique can detect a conserved 416 bp-fragment of the L1 gene that encodes RdRp (87). Identification of MRV was confirmed in all positive samples through sequencing of the L1 gene using the Sanger method (unpublished – Campalto et al.).

#### 2.3.11 Influenza A virus

##### 2.3.11.1 Molecular investigations

A 175-sample set of dogs’ and a 218-sample set of cats’ OP swabs were attempted for the detection of the Influenza A virus, using a validated screening method for Influenza A of swine origin targeting the M gene (88).

#### 2.3.12 Cowpoxvirus

##### 2.3.12.1 Molecular investigations

Cowpox virus was searched on 194 swabs collected in cats, by means of a modified rPCR protocol by Gavrilova et al. amplifying a 128 bp- fragment within the ORF D11L of Cowpox viral genome (89).

### 2.4 Statistics

The prevalence levels of all recorded pathogens were calculated; given that a sampling was adopted and that not for all pathogens the initial number of samples was reached the corresponding Wilson 95% confidence intervals were calculated in order to have a measure of the precision of the prevalence estimates. The Pearson’s chi-square test was used to explore potential associations between different methods of identifying pathogen positivity (such as molecular analysis and serology). Data were analyzed using R software (version 4.1.0) and Microsoft Excel^®^.

## 3 Results

### 3.1 Sampled population

**Figure 1:**
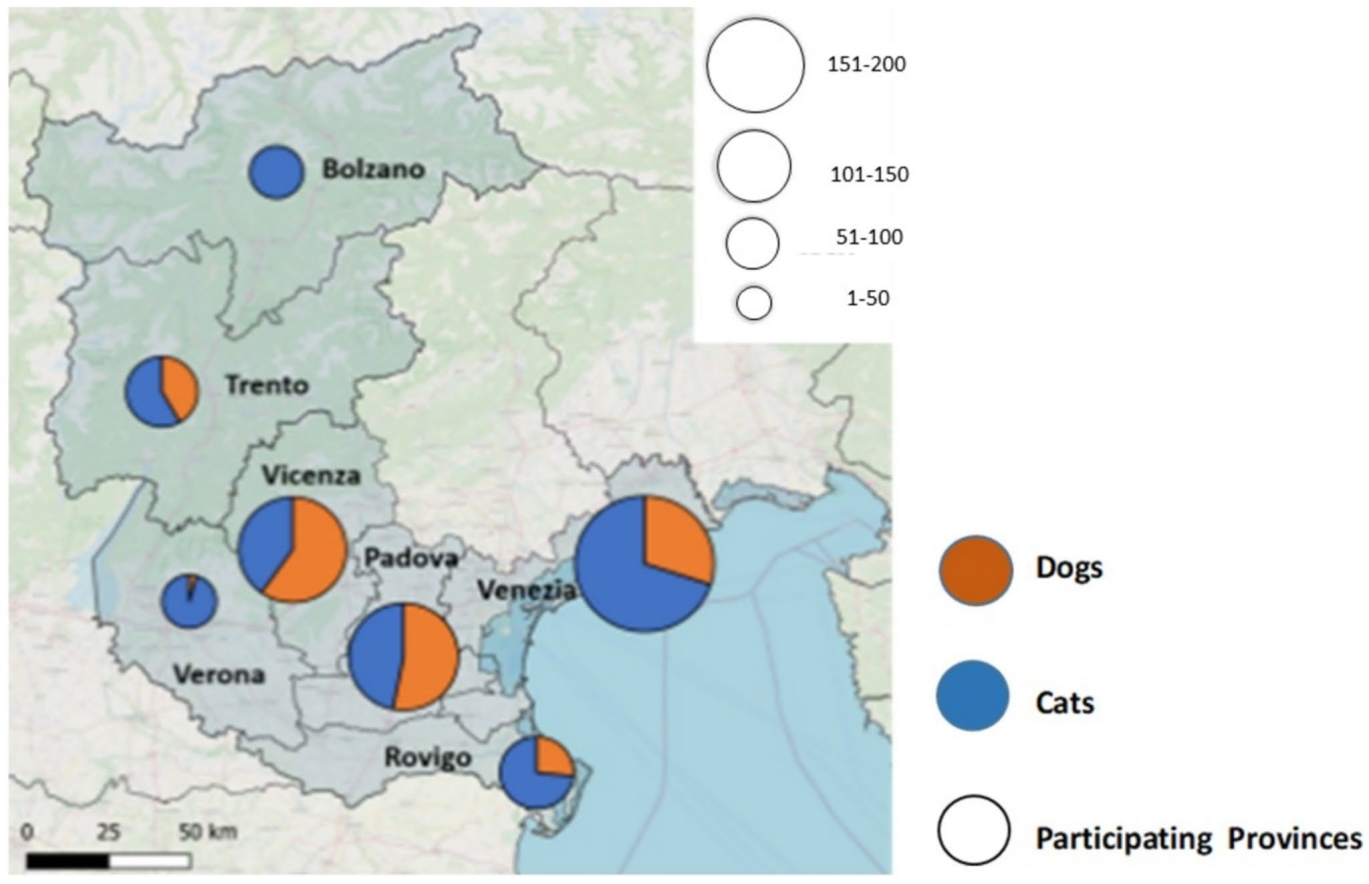
The attached graphical representation illustrates the sample distribution according to the province of origin in the North East of Italy.

Samples were collected from a total of 257 dogs and 389 cats in the period between May 2021 and September 2022 in seven provinces of North-East Italy (Bolzano and Trento in the Trentino-Alto Adige region; Padova, Rovigo, Venezia, Vicenza, and Verona in the Veneto region) (Figure 1). Epidemiological and clinical data were recorded. The animals were grouped according to their age (<1 y/o (years old), from 1 to 4 y/o, from 5 to 10 y/o, and > 10 y/o) and their breeds. The majority of the dogs included in the study were male (*n* = 164/257; 65%), whereas in the cat group, females were slightly outnumbered (*n* = 197/394; 51%). The majority of the dogs were asymptomatic at the time of sampling (87.9%), and a similar pattern was observed in the cat group (asymptomatic cats 84.6%). In some cases, the animals had complex or multiple pathologies (1.2% of dogs; 2.1% of cats). The study population demographic characteristics and epidemiological data are summarized in Table 2.

**Table 2:** Demographic information of the enrolled animals.

	Dogs (257) N (%)	Cats (389) N (%)
Sex		
Male	144 (57.1%)	139 (37.3%)
MN	20 (7.9%)	36 (9.2%)
Female	77 (30.5%)	144 (37.2%)
FN	11 (4.4%)	53 (13.6%)
n.a	5 (1.9%)	17 (4.4%)
Age (years)		
<1	62 (24.1%)	114 (29.3%)
1–4	104 (40.5%)	187 (48.1%)
5–10	43 (16.7%)	16 (4.1%)
>10	25 (9.7%)	3 (0.8%)
n.a.	23 (8.9%)	69 (17.7%)
Breed		
Crossbreed	213 (82.9%)	European shorthair 389 (100%)
Hunting dogs	22 (8.6%)	
Herding dogs	5 (1.9%)	
Molossoid dogs	17 (6.6%)	
Provinces		
Bolzano	0 (0%)	16 (4.1%)
Padova	76 (29.6%)	67 (17.2%)
Rovigo	21 (8.2%)	58 (14.9%)
Trento	30 (11.7%)	42 (10.8%)
Venezia	54 (21.0%)	131 (33.7%)
Vicenza	75 (29.1%)	53 (13.6%)
Verona	1 (0.4%)	22 (5.7%)
Clinical symptoms		
Asymptomatic	226 (87.9%)	329 (84.6%)
Respiratory	2 (0.8%)	10 (2.6%)
Gastrointestinal	6 (2.3%)	1 (0.3%)
Ectoparasites	8 (3.1%)	32 (8.2%)
Cutaneous lesions	12 (4.7%)	9 (2.3%)
Others or multiple	3 (1.2%)	8 (2.1%)

N, number of animals; n.a., not available; MN, male neutered; FN, female neutered.

### 3.2 Zoonotic pathogens

#### 3.2.1 Pathogenic *Leptospira*

No examined dogs showed any clinical signs suggestive of leptospirosis. The blood samples yielded negative results for *Leptospira* DNA, whereas the urine samples from two asymptomatic dogs tested positive (*n* = 2/257; 0.78% CI 0.08–1.25). Genotyping was attempted on the DNA of both positive cases using the MLST technique at the CNR for Leptospirosis (IZSLER). The strain *L. interrogans* serogroup Icterohaemorrhagiae ST17 was identified in the urine sample collected from an unvaccinated dog; for the second RT-PCR positive sample, taken from a vaccinated dog, the genotyping failed due to insufficient nucleic acid amount. The second dog has had a vaccination protocol in accordance with the guidelines (90) with a commercial vaccine containing *L. interrogans* serogroup Canicola serovar Portland-vere, *L. interrogans* serogroup Icterohaemorrhagiae serovar Copenhageni, *L. interrogans* serogroup Australis serovar Bratislava, *L. kirschneri* serogroup Grippotyphosa serovar Dadas. Both dogs were also positive for anti-*Leptospira* antibodies (Table 3). All the urine culture (*n* = 144) tested *Leptospira* negative.

**Table 3:** Asymptomatic *Leptospira* carrier dogs.

Case log	MAT	Real-time PCR	Bacterial isolation	Genotyping
Unvaccinated dog Male Crossbreed 1 y/o Outdoor Asymptomatic	*L. interrogans* sg Icterohaemorrhagiae serovar Icterohaemorrhagiae (titer 1:3200) *L. interrogans* sg Icterohaemorrhagiae serovar Copenhageni (titer 1:3200) *L. interrogans* sg Canicola serovar Canicola (titer 1:400)	Positive (Urine)	Negative	*L. interrogans* Icterohaemorrhagiae ST17
Vaccinated dog (*) Female Crossbreed 2 y/o Outdoor Asymptomatic	*L. kirschneri* sg Grippotyphosa serovar Grippotyphosa (titer 1:200) *L. interrogans* sg Icterohaemorrhagiae serovar Copenhageni (titer 1:100) *L. borgpetersenii* sg Sejroe serovar Hardjo (titer 1:200) *L. borgpetersenii* sg Sejroe serovar Saxkoebing (titer 1:400) *L. borgpetersenii* sg Sejroe serovar Sejroe (titer 1:100) *L. borgpetersenii* sg Ballum serovar Ballum (titer 1:100)	Positive (Urine)	Negative	Not determined

(*): Vaccine strains: *L. interrogans* serogroup Canicola serovar Portland-vere, *L. interrogans* serogroup Icterohaemorrhagiae serovar Copenhageni, *L. interrogans* serogroup Australis serovar Bratislava, *L. kirschneri* serogroup Grippotyphosa serovar Dadas.

**Figure 2:**
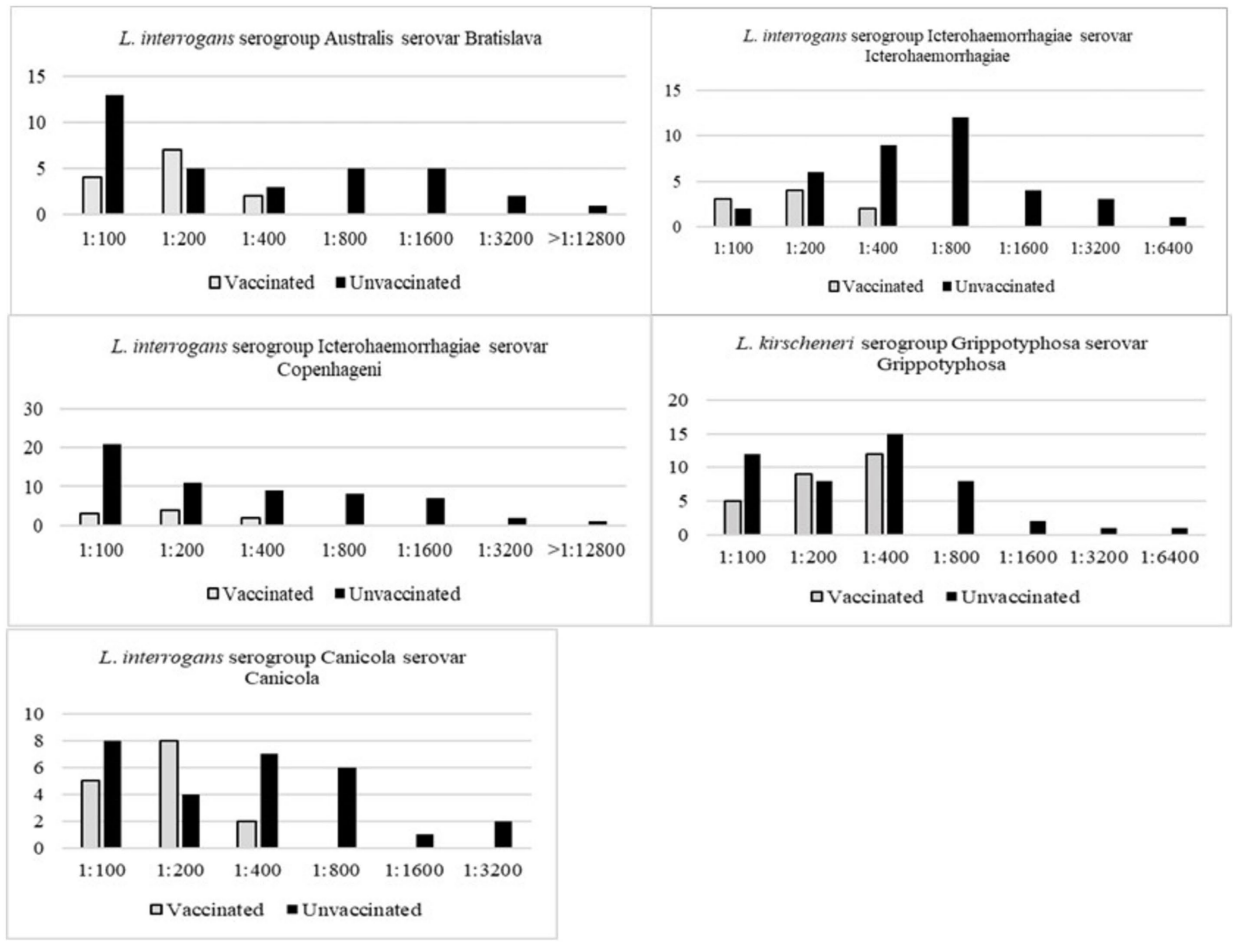
Graphical representation of serological positivity and MAT titers for serovars that have available vaccination for dogs, according to the WSAVA Guidelines (158) guidelines for the vaccination of dogs and cats—compiled by the Vaccination Guidelines Group (VGG) of the World Small Animal Veterinary Association (WSAVA) (155).

Serology for anti-*Leptospira* antibodies has an overall positivity of 44.36% (*n* = 114/257, CI 38.28–50.43%). The serological positivity was evaluated alongside data gathered through an epidemiological questionnaire. The vaccination status was evaluated for each animal, and the dogs were classified as “vaccinated” when they received a booster for leptospirosis vaccination at least 15 days prior to sampling and within the past 12 months, although absolute certainty cannot be guaranteed. Fifty animals were identified as “vaccinated” against leptospirosis, and the antigens included in vaccine formulations were registered (Table 4 and Figure 2).

**Table 4:** Number of dogs that received a full vaccination, according to the international guidelines (158) guidelines for the vaccination of dogs and cats—compiled by the Vaccination Guidelines Group (VGG) of the World Small Animal Veterinary Association (WSAVA) (155).

Vaccinated Dogs *N* = 50	Vaccine strains
34	*L. interrogans* serogroup Canicola serovar Portland-vere *L. interrogans* serogroup Icterohaemorrhagiae serovar Copenhageni *L. interrogans* serogroup Australis serovar Bratislava *L. kirschneri* serogroup Grippotyphosa serovar Dadas
10	*L. interrogans* serogroup serovar Canicola *L. interrogans* serogroup Icterohaemorrhagiae serovar Icterohaemorrhagiae *L. kirschneri* serogroup Grippotyphosa serovar Grippotyphosa
6	*L. interrogans* serogroup Canicola serovar Canicola *L. interrogans* serogroup Icterohaemorrhagiae serovar Icterohaemorrhagiae *L. kirschneri* serogroup Grippotyphosa serovar Grippotyphosa

**Figure 3:**
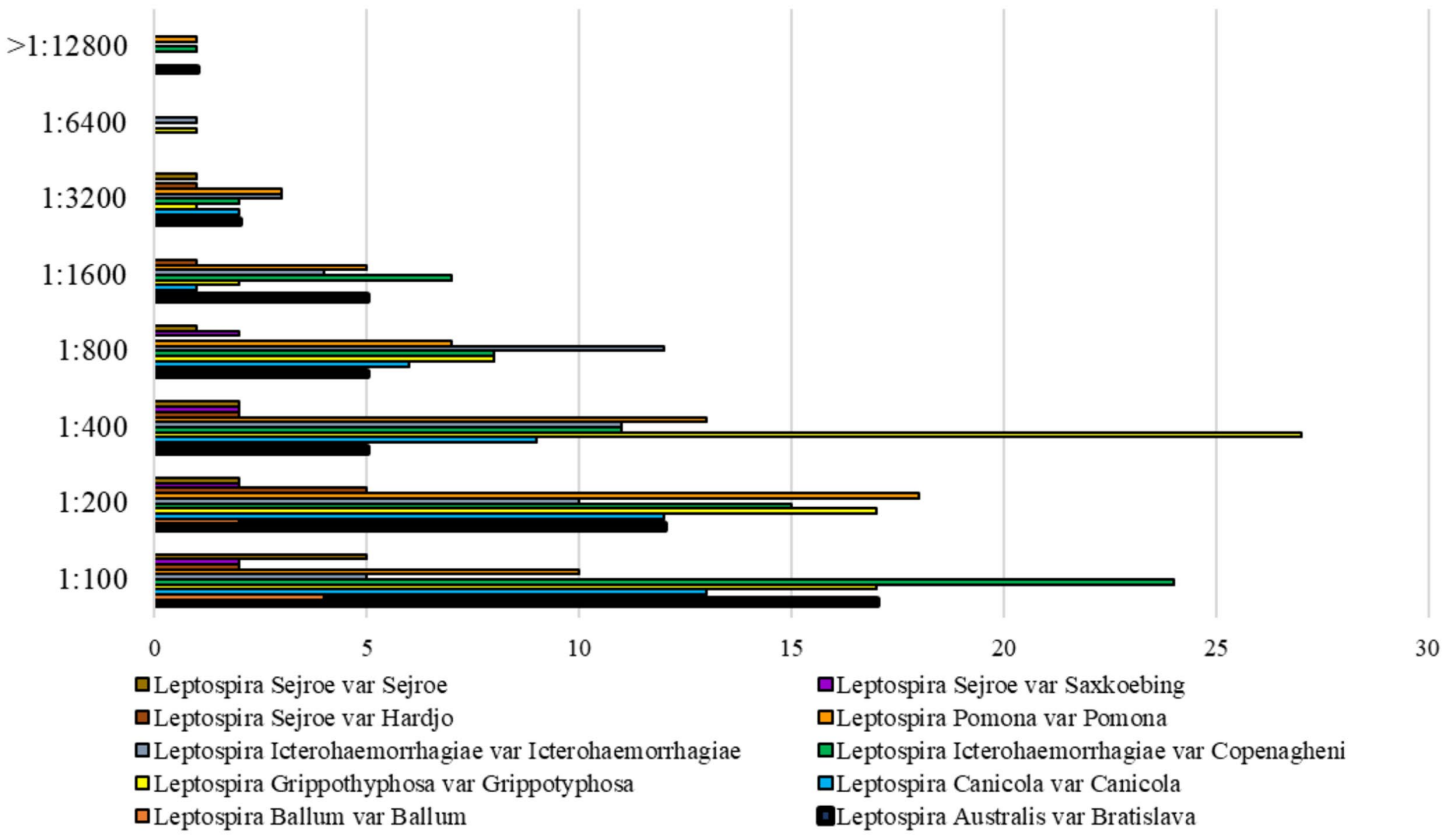
Distribution of *Leptospira* spp. MAT positivities with titer ≥1:100 of the 257 dog sera.

The overall *Leptospira* serovars detected were Grippotyphosa (*n* = 47, 18%), Copenagheni (*n* = 59, 23%), Icterohaemorrhagiae (*n* = 37, 14%), Bratislava (*n* = 34, 13%), Canicola (*n* = 28, 11%), Pomona (*n* = 57, 22%), Hardjo (*n* = 11, 4%), Saxkoebing (*n* = 8, 3%), Sejroe (*n* = 11, 4%), and Ballum (*n* = 6, 2%). No sample tested positive for *Leptospira borgpetersenii* serovar Tarassovi. Multiple reactions against two or more serovars were common. The distribution of overall serological MAT titers is depicted in Figure 3.

#### 3.2.2 Leishmania infantum

The serological positivity for anti-*Leishmania infantum* antibodies was 25% (*n* = 65/257, CI 19.98–30.61%) when using the IFAT test cut-off 1:40. Applying the IFAT ≥1:160 threshold reduces the prevalence by 3.89% (*n* = 10/257, CI 1.53–6.26%).

#### 3.2.3 *Bartonella henselae* and *Bartonella clarridgeiae*

*Bartonella* spp. DNA reported a prevalence of 25.91% (*n* = 100/386; CI 21.54–30.28%): *Bartonella henselae* was the most common species (*n* = 52; 13.47% CI 10.42–17.24%), followed by *Bartonella clarridgeiae* (*n* = 28; 7.25% CI 5.07–10.28%), while co-infections *Bartonella henselae* – *Bartonella clarridgeiae* were reported in 20 of 387 cats (5.18% CI 3.38–7.87%).

Serological tests for *Bartonella henselae* reported a prevalence of 70.18% (*n* = 273/389; CI 66.63–74.73%) using the IFAT cut-off test (≥1:64). Applying IFAT ≥1:128 the prevalence decreased to 50.4% (*n* = 196/389; CI 45.42–55.35%).

Of the 386 cats tested by both molecular and serological methods, the results for 93 subjects (24.09%; CI 19.83–28.36%) showed both *Bartonella* DNA detection and seropositivity. Serological test results were compared with molecular test results to determine the distribution of positivity for both *Bartonella* species. No statistical significance was reported among the positivity to the IFAT titer and the *Bartonella* species detected with molecular analysis (*p* > 0.05) (Table 5).

**Table 5:** Distribution of molecular positivity for *Bartonella* spp. and IFAT titers.

Molecular analysis (PCR)	IFAT *B. henselae*				
	1:64	1:128	1:256	>1:256	Total
*Bartonella clarridgeiae*	6 (40.0%)	5 (38.5%)	5 (21.7%)	11 (26.2%)	27
*Bartonella henselae*	7 (46.7%)	4 (30.8%)	13 (56.5%)	22 (52.4%)	46
*B. clarridgeiae + B. henselae*	2 (13.3%)	4 (30.8%)	5 (21.7%)	9 (21.4%)	20
Total	15 (16.1%)	13 (14.0%)	23 (24.7%)	42 (45.2%)	93

#### 3.2.4 Antibiotic-resistant bacteria

The occurrence of extended-spectrum *β*-lactamase (ESBL)-producing *Enterobacteria*, *Pseudomonas* spp., vancomycin-resistant *Enterococci* (VRE), and methicillin-resistant coagulase-positive *Staphylococci* (MRCPS) was evaluated on 646 animals, comprising 389 cats and 257 dogs. All samples tested negative for MRCPS and VRE, while the detection of ESBL-producing *Enterobacteria* and *Pseudomonas* spp. reported overall prevalence of 7.2% in cats (*n* = *n* = 28/389), distributed as follow: *n* = 21; 39.29% CI 21.20–57.38, *n* = 7; 25% CI 8.96–41.04, respectively. In dogs, the prevalence of ESBL-producing *Enterobacteria* and *Pseudomonas* spp. reported *n* = 81/275 (31.52%) distributed as follow: *n* = 38; 46.91% CI 36.05–57.78 and *n* = 43; 53.09% CI 42.22–63.95, respectively. Among the dogs positive for *Pseudomonas* spp., a large number reported positivity for *Pseudomonas aeruginosa* (*n* = 41; 50.62% CI 39.73–71.51).

#### 3.2.5 Brucella canis

Serological test for anti-*Brucella canis* antibodies reported overall prevalence of 1.95% (*n* = 5/257: CI 0.26–3.63%). *Brucella canis* DNA was not found in any blood or urine samples, nor did the isolation detect any positivity (Table 6).

**Table 6:** *Brucella canis* serology and bacterial culture (blood and urine) in dogs.

Case log	mSAT	CFC	IF	Bacterial culture
Male Neutered Crossbreed 1 y/o Asymptomatic	1:40	1:20	1:40	Negative
Male Rottweiler 5 y/o Testicular hypoplasia	1:20	1:10	1:80	Negative
Female Crossbreed Asymptomatic	1:20	1:40	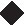	Negative
Male Crossbreed 7 y/o Asymptomatic	1:40	1:40	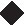	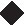
Male German Shepherd 6 y/o Weight loss Haematuria Diarrhea PU/PD	1:40	1:10	1:80	Negative

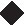
, Not performed.

#### 3.2.6 Zoonotic intestinal parasites and dermatophytes

The coprological examination showed a higher prevalence of parasites in cats (*n* = 72/143; 50.35% CI 42.15–58.54%) than in dogs (*n* = 35/177; 19.77% CI 13.91–25.64%), mostly in young subjects (1–5 y/o) in both species. The most reported parasites groups were intestinal nematodes (ancylostomes, ascarids, capillariae and trichuridae), coccidia (most prevalent in dogs) and *Taenia taeniformes* identified in only three cats (*n* = 3/143; 2.10% CI 0.00–4.45). Apart from tapeworms, all other fecal samples from both dogs and cats tested negative for *Echinococcus* species. The prevalence of dermatophytes was very low overall (*n* = 4/646; 0.6% CI 0.01–1.22): three cats were positive, reporting *Microsporum canis* in a single cat and *Nannizia gypsea* in two (*n* = 3/389; 0.77% CI 0.00–1.64), and one dog was positive for *Microsporum canis* (*n* = 1/257; 0.93% CI 0.00–1.15).

#### 3.2.7 *Capnocytophaga* spp.

The bacteriological culture tested positive for *Capnocytophaga* sp. in 12.45% of dogs (*n* = 32/257; CI 8.41–16.48%) and 5.9% of cats (*n* = 23/389; CI 3.6–8.9%). Molecular analysis reported positivity in 82.5% dogs (*n* = 212/257; CI 77.4–86.6%) and 64.8% cats (*n* = 252/389; CI 59.9–69.4).

#### 3.2.8 SARS-CoV-2

The overall seroprevalence reported in the dogs’ population was 3.5% (*n* = 9/257; CI 1.25–5.75), and no positivity was reported both in OP and R swabs with regard to molecular analysis. After molecular investigation on 389 feral cats, two OP swabs resulted positive (0.5% *n* = 2/389; CI 0.00–1.22) and the overall seroprevalence in cats was 0.8% (*n* = 3/389; CI 0.00–1.64%). One cat reported positivity for SARS-CoV-2 molecular assay and serum antibodies simultaneously.

#### 3.2.9 Hepatitis E – influenza – norovirus – cowpoxvirus

The emergent zoonotic agents were investigated in dogs and cats using direct pathogen genetic material detection methods. No molecular positivity was found in either animal species for the aforementioned agents (Table 7). The serological prevalence of HEV in the enrolled animals was also investigated and no positivity was reported.

**Table 7:** Hepatitis E – Influenza – Norovirus – Cowpoxvirus samples analyzed per species and per analysis.

Analysis	Dogs	Cats
Hepatitis E virus	0/176 (0%; CI 0–2.07%)	0/142 (0%; CI 0–2.56%)
Hepatitis E serology	0/257 (0%; CI 0–1.42%)	0/389 (0%; CI 0–0.94%)
Influenza A virus	0/175 (0%; CI 0–2.08%)	0/218 (0%; CI 0–1.67%)
Norovirus	0/193 (0%; CI 0–1.89%)	0/297 (0%; CI 0–1.23%)
Cowpoxvirus	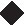	0/194 (0%; CI 0–1.88%)

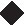
, Not performed.

#### 3.2.10 Mammalian orthoreovirus

Eleven cats tested positive for MRV RNA detection (*n* = 11/388; 2.83% CI 1.59–5.01%): seven cats tested positive in R swabs, two cats in OP swabs, and two cats reported molecular positivity both in R and OP swabs. One dog (*n* = 1/257; 0.38% CI 0.07–1.15%) tested positive on OP swab. Positivity for MRV was confirmed in six cat samples through Sanger sequencing of the L1 gene from animals sampled in the same session.

The positive results in cats were detected in young animals ranging from 1 to 2 years old and originated from three cat colonies: the Bozen province (*n* = 5), Padua (*n* = 4), and Venice (*n* = 2).

#### 3.2.11 Rotavirus A

Two mixed-breed female dogs (both 3.5 years old) from the provinces of Padua and Trento were positive (*n* = 2/255; 0.78% CI 0.01–1.87%). The dogs did not show any gastrointestinal sign at the sampling. Thirteen cat tested positive (*n* = 13/389; 3.27% CI 1.52–5.01%). These animals were young cats aged between 6 months and 2 years, collected in colonies located in the provinces of Venice (*n* = 7), Padua (*n* = 2), Vicenza (*n* = 3), and Rovigo (*n* = 1).

The results of the investigation on the prevalence of emerging, neglected and known zoonotic agents are summarized in Table 8.

**Table 8:** List of zoonotic pathogens investigated during the health monitoring of dogs and cats in shelters (*n* = 646), type of analysis performed and biological material sampled.

Pathogen	Analysis	Biological sample	Dogs (257) N (%)				Cats (389) N (%)			
			Negative	Positive	Total	95% CI Prevalence	Negative	Positive	Total	95% CI Prevalence
*Leptospira* spp.	MAT	Serum	146	114	257	44.36 (38.28–50.43)	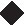	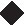	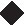	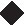
	Real time PCR	Urine	255	2	257	0.78 (0.08–1.25)	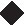	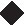	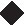	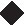
		Blood	144	0	144	0.0 (−)	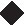	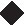	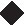	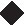
	Isolation	Urine	144	0	144	0.0 (−)	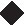	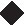	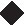	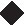
*Leishmania infantum*	IFAT	Serum	195	65	257	25.0 (19.98–30.61)	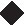	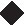	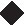	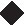
Bacteria ESBL	Cultural	Rectal swab/feces; Oral swabs	176	81	257	31.52 (25.84–37.20)	361	28	389	7.2 (4.63–9.7)
Bacteria MRCPS	Cultural	Rectal swab/feces; Oral swabs	257	0	257	0.0 (−)	389	0	389	0.0 (−)
Bacteria VRE	Cultural	Rectal swab/feces; Oral swabs	257	0	257	0.0 (−)	389	0	389	0.0 (−)
*Brucella canis*	mSAT	Serum	252	5	257	1.95 (0.26–3.63)	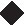	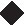	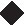	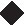
Dermatophytes	Cultural	Hair/crust/skin lesions	254	1	257	0.93 (0.00–1.15)	386	3	389	0.77 (0.00–1.64)
Nematode and Coccidia	Flotation	Feces	142	35	177	19.77 (13.91–25.64)	71	72	143	50.35 (42.15–58.54)
Taenia spp.	Flotation	Feces	177	0	177	0.0 (−)	140	3	143	2.10 (0.00–4.45)
*Capnocytophaga* sp.	Cultural (MALDI-TOF)	Oral swabs	225	32	257	12.45 (8.41–16.48)	366	23	389	5.9 (3.6–8.9)
	Real time PCR	Oral swabs	45	212	257	82.5 (77.4–86.6)	139	252	389	64.8 (59.9–69.4)
*Bartonella henselae*	IFAT	Serum	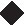	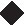	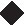	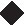	116	273	389	70.18 (66.63–74.73)
*Bartonella* sp.	Real time PCR	Blood	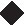	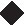	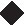	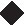	286	100	386	25.91 (21.54–30.28)
Norovirus	Real time RT-PCR/ Sanger sequencing	Rectal swabs/feces	193	0	193	0.0 (−)	297	0	297	0.0 (−)
Cowpox virus	Real time PCR	Hair/crust/skin lesions	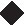	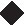	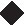	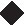	194	0	194	0.0 (−)
Hepatitis E	Real time RT-PCR	Rectal swabs/feces	176	0	176	0.0 (−)	142	0	142	0.0 (−)
	ELISA	Serum	275	0	257	0.0 (−)	389	0	389	0.0 (−)
Influenza A virus	Real time RT-PCR	Rectal swab/feces; Oral swabs	175	0	175	0.0 (−)	218	0	218	0.0 (−)
Rotavirus A	Real time RT-PCR	Rectal swabs/feces	253	2	255	0.78 (0.01–1.87)	376	13	389	3.27 (1.52–5.01)
Mammalian Orthoreovirus	End-point RT-PCR/ Sanger sequencing	Rectal swabs/feces	256	1	257	0.38 (0.07–1.15)	377	11	388	2.83 (1.59–5.01)
Coronavirus (SARS-CoV-2)	Real time RT-PCR	Rectal swab/feces Oral swabs	257	0	257	0.0 (−)	387	2	389	0.51 (0.00–1.22)
	ELISA	Serum	248	9	257	3.5 (1.25–5.75)	386	3	389	0.8 (0.02–1.64)

ESBL, Extended-Spectrum Beta-Lactamases bacteria; MRCPS, Methicillin-resistant coagulase positive staphylococci; VRE, Vancomycin resistant enterococci; MAT, Microscopic agglutination test; IFAT, indirect fluorescent antibody test; ELISA, enzyme-linked immunosorbent assay; RT-PCR, Reverse transcription polymerase chain reaction; PCR, polymerase chain reaction; MALDI-TOF, matrix-assisted laser desorption/ionization time-of-flight mass spectrometry.

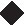
, Not performed.

## 4 Discussion

Zoonoses, despite being recognized for centuries, have recently garnered heightened attention, particularly in the post-COVID-19 era. Consequently, the prevention and control of zoonotic diseases must be revised by focusing on the complex interaction between humans, animals, and the ecosystem through the One Health approach. In a context of routinely health monitoring of kennel dogs and free-roaming cats in North-East Italy, this study updates about the epidemiology and the circulation of known, neglected, potential, emerging and re-emerging zoonoses among the sheltered dogs and cats. The updated acknowledgement of the presence and prevalence of zoonotic agents is pivotal for a correct optimization of any surveillance action (91). Companion animal shelters present a challenge in balancing animal health and welfare, staff organization and training, and economic resources. It is critical to implement effective surveillance for infectious or zoonotic agents in companion animal kennels, dog and cat colonies or stray communities where animals of different ages, breeds and origins, often with poorly known medical histories, live together in close contact (92–94).

The present results confirmed the data about leptospirosis as previously reported in dogs and wild animals in the North-East Italy (2, 3, 95, 96), and that except for *L. interrogans* serogroups Pomona and Sejroe, the serovars most commonly found were those already targeted by vaccines commercially available, specifically serogroups Grippotyphosa, Icterohaemorrhagiae, Bratislava, and Canicola. Animals with the highest MAT titers (≥1:800) were typically those that had not received vaccination against the specific serogroup. As published data is limited regarding the incidence of naturally occurring leptospirosis in fully vaccinated dogs (97, 98), it is interesting to report that two apparently healthy dogs, one of which received regular vaccination against leptospirosis, had asymptomatic shedding of *Leptospira* (urine). The outstanding information about the carrier dogs is that they were clinically healthy, outdoors and young (1 y/o and 2 y/o). This finding thus indicates the necessity of implementing monitoring protocols for kennel resident animals or those newly admitted to the shelters and of emphasizing the importance of operators’ observance of biosecurity measures.

The seroprevalence data for leishmaniosis were consistent with recent studies conducted in the same areas (6, 99) indicating a significant prevalence of exposure to the pathogen (6, 8, 16, 100). The majority of the sampled animals were asymptomatic, and for a small percentage data on antiparasitic treatment was available, mainly oral medication (11.15%; CI 7.33–14.98). Although most positive dogs reported low IFAT titers (from 1:40 to 1:80), indicating contact with the pathogen rather than clinical active infection, it is pivotal to acknowledge that the North-East Italy includes areas of gradually increasing endemicity (99, 101). This situation highlights the necessity of continuous monitoring, not only to safeguard animal health but also to prevent zoonotic risks (102). In addition, our results confirm that animal shelters play a critical role in identifying positive subjects traveling from highly endemic regions (i.e., southern Italy) (103, 104).

Furthermore, this study reported a significant prevalence of *Bartonella* spp., exceeding that which was previously reported (5, 13, 15, 105), both for active infection (*Bartonella* spp. DNA) and serological positivity. Only seven cats were PCR-positive for *B. henselae*, but tested negative in serology, suggesting recent infection/reinfection. In addition, by comparing the positivity between the molecular and serological tests, our results indicate that the IFAT analysis for *B. henselae* gave positive results in animals where there was evidence of only *B. clarridgeiae* DNA. This finding evokes serological cross-reactivity among various *Bartonella* species, which have been reported also for other agents and bacterial species (106–108). This aspect can suggest that the serology for *Bartonella* sp. could be a useful screening tool in the large population; however, as with human medicine (109), molecular analysis is the confirmatory test. Most of the cats positive for *Bartonella* species on direct DNA testing were also positive on serology, although statistical significance was not reported between the *Bartonella* species identified by molecular analysis and IFAT titers. The presence of the arthropod vector *Ctenocephalides felis* is closely associated with *Bartonella* sp. infection in cats and free-living or colony animals are frequently re-exposed, as they do not receive regular anti-parasite prophylaxis. In felines, the infection can have a host-adapted-reservoir course showing either an asymptomatic or a paucisymptomatic form (15, 74, 110). Conversely, in humans, the infection can exhibit severe forms of the disease, especially in young or immune-compromised people (111–116). Hence, it is high-priority to ascertain the latest prevalence and spread of the infection, and effectively communicate the associated zoonotic risk to companion animals’ shelters operators and the general public (110, 117). Treatment of this infection in feral cats with drugs is not feasible and may prove ineffective. Instead, preventive measures must center on flea prophylaxis to diminish the likelihood of infection in cats (117–121). In addition, training for health and ethology professionals reduces the probability of exposure to bite and scratch wounds.

The antibiotic-resistant bacteria are significant sources of hospital-acquired infections (122–124), reported low prevalence within the studied population: no methicillin-resistant coagulase-positive Staphylococci (MRCPS) or vancomycin-resistant Enterococci (VRE) were isolated. According to previous studies, the prevalence of ESBL-producing Enterobacteria and *Pseudomonas aeruginosa* was higher in dogs than in cats. The significant number of positive subjects, mostly asymptomatic, highlights the need to implement the surveillance of this pathogen to prevent the emergence and spread of multidrug-resistant strains, particularly in shelter and kennel situations (24, 59, 125–127). The low prevalence of antibiotic resistance among animals in shelters and/or free-living cats may be attributed to their limited exposure to antibiotics (128, 129), as well as some of them not living constantly close to humans. Additionally, veterinary professionals responsible for the facilities follow antibiotic stewardship guidelines, contributing to the low prevalence observed (130, 131). Nevertheless, the aforementioned is a dynamic circumstance, and the findings imply that surveillance of these microorganisms should be considered, especially in scenarios that might promote their proliferation such as overcrowding, inadequate hygiene, and recurring bacterial illnesses.

*Brucella canis* is a zoonotic pathogen considered to be emerging because of the movement of animals, including trade and legal/illegal transfer, from countries where this disease is more prevalent or endemic (132, 133). The diagnosis of *Brucella canis* remains challenging owing to the inadequacy of current diagnostic methods; indeed, the diagnosis demands multiple tests to be run in parallel, including different serological methods, molecular analysis, and bacterial isolation (75). Furthermore, the bacterium’s biological and pathophysiological characteristics complicate diagnostic accuracy (134), and uncertain clinical manifestations may lead to underdiagnosed of this disease. In this study, none of the five serological positivities were followed by confirmation by direct pathogen detection analysis. Specifically, we reported a case of clinically asymptomatic dog (7-year-old mixed-breed male) with anti-*Brucella canis* mSAT antibody titer of 1:40 and concurrently positive for *Trichuris* spp. at the coprological examination. One month later, the dog was retested using serological methods and was found to be seronegative. The second case involved a Rottweiler, a male dog 5 years old and reported both testes reduced in size. Serology reported mSAT 1:20, CFT 1:10 and IF 1:80. However, bacterial isolation proved negative. After a month, this dog was retested, reporting mSAT 1:20, CFT negative, IF 1:40, negative bacterial culture (in both urine and blood), and borderline *Brucella* spp. PCR positivity. Indeed, our results reported a low overall seroprevalence for anti-*Brucella canis* antibodies and no positivity was confirmed on direct research of the pathogen. Nevertheless, there is a potential zoonotic risk to consider and communicate. An intensified collaboration with the veterinary medical professionals would be desirable to include this pathogen more frequently in the list of differential diagnoses, particularly in clinical pictures consistent with an acute or chronic infection caused by this microorganism (26, 28, 135).

Gastrointestinal parasites reported a high prevalence in both investigated species, especially in cats, according with recent literature (16, 101, 120, 136). Possible reasons for the lower occurrence of parasitosis found in dogs include increased control of confined dogs and more frequent use of anti-parasite treatment in refuge areas and kennels. Cats that have access to large outdoor areas are at a higher risk of being exposed frequently to environments contaminated with intestinal helminth eggs and consequently at a greater risk of re-infestation (120). The majority of parasite species identified in cats belong to the nematode family, are specific to certain species and pose limited zoonotic potential, mainly in ‘fragile’ categories or in children or immunocompromised people (1, 137–140). In addition, our study highlights that parasites in dogs and cats may require a specific prevention and control protocol, as these infestations can lead to acute, chronic and long-term health problems that affect shelter management from a medical, economic and managerial perspective (64, 120, 141). Notably, this study reported reassuring data about the prevalence of *Echinococcus* among the sheltered or free-roaming companion animals, which yielded no positive results in the investigated area. However, given the dangerous nature of the parasite (94, 142) and its prevalence among wildlife (11, 78), it may be useful to include this agent within a screening panel for zoonotic pathogens surveillance, or a specific and systematic prophylaxis. Additionally, it is recommended that training programme are provided for kennel and cat colony operators, as well as auxiliary staff in biosecurity, to reduce the risk of animal infestation (143). Differently to common perception, cases of dermatophytosis were rarely reported among our study population. The infected animals were either immunocompromised or living in overcrowded conditions. This result is particularly noteworthy, as previous studies have traditionally associated highly populated areas like shelters and catteries with a higher prevalence of dermatophytosis (144), whether it be through asymptomatic carriers or clinical signs, especially in cats (10, 17, 145). Low positivity to dermatophytes may be consistent with good and efficient management of the facilities in which the animals are housed. Moreover, we want to highlight that Italian legislation protects free-living feral cats and does not provide for close catteries except for health reasons and for a short time (Italian Law 281/1991; Agreement of 6 February 2003 between the Ministry of Health, the regions and the autonomous provinces of Trento and Bolzano ‘on the welfare of pets and pet-therapy’; D. Lgs n°134–05/08/2022). Therefore, the cat population lives in free colonies and crowding is limited.

The potential or emerging zoonotic viruses, such as Hepatitis E virus, Influenza A, Norovirus, and Cowpoxvirus, were also investigated. Although no positive results were recorded, it is important to note that the majority of the animals sampled in this study did not show any clinical symptoms. Therefore, it is recommended to include among the differential diagnoses these agents, when managing animals with medical history, clinical presentation and epidemiological data, suggestive of zoonotic diseases related to the aforementioned microorganisms.

A different scenario has opened up for two *familiae* of viruses, for which positivities have been observed, in particular in cats: Mammalian Orthoreovirus (MRV) and Rotavirus A.

MRV is an emerging zoonotic agent (146) that is responsible for causing gastrointestinal diseases in both humans and animals. Although some positive cases have been found in wildlife in areas adjacent to those covered in this study (147, 148), no data on the prevalence of this microorganism among dogs and cats in Italy have been recorded to date. This study reported positive samples in cats, where the viral RNA was detected primarily in fecal samples, although both OP swabs and feces yielded two MRV-positive cats. It is noteworthy that the positive cats were part of the same feline colony and were sampled in a single session, possibly indicating a cluster outbreak as previously reported in other animal species (147, 148). The Rotavirus genus is classified into eight serogroups denoted as A to H, with four of them (A, B, C and H) having the potential to cause diseases in humans (52, 86, 149). Significantly, serogroup A accounts for over 90% of cases associated with gastrointestinal illnesses in people (53, 150–152). According to previous studies (93, 152), our results reported that cats from the same feline colony exhibited positive results during the same sampling period. Unfortunately, the clinical features for both MRV and Rotavirus A are difficult to be documented in free-living cats.

During the SARS-CoV-2 pandemic, research has aimed to investigate the role of dogs and cats in sheltered housing (82). Among the positive animals, dogs exhibit the highest serological positivity, possibly due to their higher level of interaction with humans in sheltered housing environments. On the other hand, PCR positivity was recorded in two cats, confirming the higher receptivity to the infection of this species. Details of this investigation have been already published (82). Due to its emergence, zoonotic nature, and virus characteristics, it is crucial to adhere to the guidelines and preventive measures proposed by both international and national institutions,^2^^,^^3^ eventually prompt screening for zoonotic diseases is imperative.

Of the emerging zoonotic agents, *Capnocytophaga* spp. (*C*. spp) is a microorganism characterized as commensal bacterium. Some serotypes, mainly belonging to the species *C. canimorsus*, can lead to infections in humans, particularly in immunocompromised patients (18, 20, 153, 154). Several species of *Capnocytophaga* spp. have been reported in humans (*C. ochracea, C. sputigena, C. gingivalis*), while others have been described in animals (*C. cynodegmi, C. canimorsus, C. canis, C. felis*) (21, 80). Taxonomic classification may rapidly evolve as this is a recently discovered microorganism and there could be unknown species. Infection is transmitted through contact with infected dogs and cat’s saliva or by being bitten (55). This study examines the presence of these emerging pathogens with zoonotic potential in the oral flora of companion animals through microbiological and molecular techniques. The two methods yield different outcomes, but neither of them can easily differentiate species and serotype: while the bacterial culture identifies the living organism, the rPCR detects the genetic material. Our findings confirm the presence of the microorganism in both dogs and cats; however, due to limitations in diagnostic methods and a lack of knowledge regarding the bacteria’s pathogenicity and biological behavior, an accurate diagnostic screening protocol cannot yet be defined. In front of outstanding *C.* spp. prevalence (82.5% in dogs and 64.8% in cats), the real risk to enter in contact with a dog or cat carrier of dangerous strains is probably extremely low. The results have enabled the authors to implement identification methods, but further in-depth studies are still underway. Considering the potential for the bacteria to spread to humans, particularly immunocompromised persons, it would be useful to implement collaboration with physicians, so it will be possible to gather extensively data and information on the bacterial strains that cause infections in humans.

Previous studies highlight the need of implementing communication and awareness programmes targeted toward the general public on the subject of zoonotic risk in the context of human-pet interaction (57, 58, 91, 141, 155). Recently, a study conducted in the Northeast of Italy, highlighted the ongoing need to enhance owners’ understanding of zoonoses affecting their pets and also the protective role of vaccines (156). This emphasizes the importance of adopting behaviors that promote the preservation of both the health and welfare of animals and humans alike. Furthermore, as previously reported (58, 63, 65, 157), there is a clear requirement to implement mechanisms that support the ongoing training of all companion animals shelter operators, thereby facilitating the dissemination and implementation of guidelines and best practices across the remit of each operator’s activities.

The collected data may help to prioritize zoonotic diseases, by implementing the periodical update, considering a shared algorithm that includes various parameters, such as the current and potential spread of the zoonotic agent, the severity of the infection in humans, the availability of therapeutic treatment, the probability of contracting it for operators and the public (59). The absence of specific and continuous training programmes for kennel operators or volunteers that cover topics such as infectious agents, biosecurity protocols, and basic ethological knowledge can lead to low or incorrect perception of zoonotic risk. In addition, the awareness about emergent or neglected zoonosis among the general population is usually scarce. A future perspective should be the implementation of sheltered companion animals’ health monitoring and *ad hoc* prophylactic programmes, as well as encouraging correct behavior from medical and veterinary doctors, hospital staff and citizens, as important outputs for recognizing possible emergent or neglected zoonoses. In conclusion, we want to stress the need of communication programmes in a One Health view about zoonoses and pets, and harmonized guidelines and operational protocols for better management and health approaches in dogs and cats in shelters and feline colonies. In addition, encouraging correct behavior both of operators within hospital facilities and of citizens in their daily lives, are also desirable outputs in the medium and long term to control the spread of these pathogens.

## 5 Conclusion

This study investigated the epidemiology of known, neglected, potential, emerging and re-emerging zoonotic agents. The findings yielded data on the prevalence of zoonotic diseases in the population of animals admitted to shelters, as well as integrated medical and organizational management aspects. Prevalence data confirms the incidence of several known zoonoses and sheds light on agents that are rare or only potentially zoonotic, such as MRV and Rotavirus A. This research emphasizes the necessity for more understanding on the dissemination of the emergent and neglected microorganisms and their potential for zoonotic transmission, which is presently unconfirmed, especially in contexts of human-animal interaction.

## Data Availability

The raw data supporting the conclusions of this article will be made available by the authors, without undue reservation.

## Footnotes

^1^updated 31/12/2023, https://www.salute.gov.it/portale/caniGatti/dettaglioContenutiCaniGatti.jsp?lingua=italiano&id=280&area=cani&menu=abbandono, accessed in date: 15/07/2024.

^2^https://www.cdc.gov/coronavirus/2019-ncov/animals/pets.html

^3^https://www.woah.org/fileadmin/Home/MM/EN_Factsheet_SARS-CoV-2.pdf

## References

[1] Chomel BB. Emerging and re-emerging zoonoses of dogs and cats. Animals. (2014) 4:434–45. doi: 10.3390/ani4030434 26480316 PMC4494318

[2] Bertasio C Boniotti MB Lucchese L Ceglie L Bellinati L Mazzucato M. Detection of new Leptospira genotypes infecting symptomatic dogs: is a new vaccine formulation needed? Pathogens. (2020) 9:484. doi: 10.3390/pathogens9060484 32570803 PMC7350335

[3] Tagliabue S Figarolli BM D’Incau M Foschi G Gennero MS Giordani R. Indagine sierologica sulla presenza di Leptospira spp. in Italia: Dati nazionali 2010-2011. Vet Ital. (2016) 52:129–38. doi: 10.12834/VetIt.58.169.2, PMID: 27393874

[4] Costa ACTRB Colocho RAB Pereira CR Lage AP Heinemann MB Dorneles EMS. Canine leptospirosis in stray and sheltered dogs: a systematic review. Anim Health Res Rev. (2022) 23:39–58. doi: 10.1017/S1466252321000190 35726571

[5] Spada E Canzi I Baggiani L Perego R Vitale F Migliazzo A. Prevalence of Leishmania infantum and co-infections in stray cats in northern Italy. Comp Immunol Microbiol Infect Dis. (2016) 45:53–8. doi: 10.1016/j.cimid.2016.03.001 27012922 PMC7132376

[6] Morosetti G Toson M Trevisiol K Idrizi I Natale A Lucchese L. Canine leishmaniosis in the Italian northeastern Alps: a survey to assess serological prevalence in dogs and distribution of phlebotomine sand flies in the Autonomous Province of Bolzano - South Tyrol, Italy. Vet Parasitol Reg Stud Rep. (2020) 21:100432. doi: 10.1016/j.vprsr.2020.100432 32862903

[7] Ramos RAN Giannelli A Ubirajara-Filho CRC Ramos CADN Betbder D Bezerra-Santos MA. Vector-borne pathogens in dogs from areas where leishmaniosis is endemic. Vet Parasitol. (2022) 32:100746. doi: 10.1016/j.vprsr.2022.100746, PMID: 35725105

[8] Elmahallawy EK Zanet S Poggi M Alsharif KF Agil A Trisciuoglio A. Feline leishmaniosis in northwestern Italy: current status and zoonotic implications. Vet Sci. (2021) 8:215. doi: 10.3390/vetsci8100215 34679045 PMC8539510

[9] Spada E Perego R Vitale F Bruno F Castelli G Tarantola G. Feline Leishmania spp. infection in a non-endemic area of northern Italy. Anim Open Access J MDPI. (2020) 10:817. doi: 10.3390/ani10050817 PMC7278790 32397321

[10] Iorio R Cafarchia C Capelli G Fasciocco D Otranto D Giangaspero A. Dermatophytoses in cats and humans in Central Italy: epidemiological aspects. Mycoses. (2007) 50:491–5. doi: 10.1111/j.1439-0507.2007.01385.x 17944712

[11] Obber F Celva R Da Rold G Trevisiol K Ravagnan S Danesi P. A highly endemic area of Echinococcus multilocularis identified through a comparative re-assessment of prevalence in the red fox (*Vulpes vulpes*), Alto Adige (Italy: 2019-2020). PLoS One. (2022) 17:e0268045. doi: 10.1371/journal.pone.0268045 35511816 PMC9070940

[12] Massolo A Valli D Wassermann M Cavallero S D’Amelio S Meriggi A. Unexpected Echinococcus multilocularis infections in shepherd dogs and wolves in south-western Italian Alps: a new endemic area? Int J Parasitol Parasites Wildl. (2018) 7:309–16. doi: 10.1016/j.ijppaw.2018.08.001 30175043 PMC6115541

[13] Latrofa MS Iatta R Toniolo F Furlanello T Ravagnan S Capelli G. A molecular survey of vector-borne pathogens and haemoplasmas in owned cats across Italy. Parasit Vectors. (2020) 13:116. doi: 10.1186/s13071-020-3990-x 32312323 PMC7171850

[14] Ebani VV Nardoni S Maestrini M Perrucci S Mancianti F. Serological survey on the occurrence of Rickettsia spp., Neospora caninum, Bartonella henselae and toxoplasma gondii in cats from Tuscany (Central Italy). Anim Open Access J MDPI. (2021) 11:1842. doi: 10.3390/ani11061842 PMC8234574 34205734

[15] Brunetti E Fabbi M Ferraioli G Prati P Filice C Sassera D. Cat-scratch disease in northern Italy: atypical clinical manifestations in humans and prevalence of Bartonella infection in cats. Eur J Clin Microbiol Infect Dis. (2013) 32:531–4. doi: 10.1007/s10096-012-1769-5, PMID: 23132688

[16] Traversa D Di Cesare A Simonato G Cassini R Merola C Diakou A. Zoonotic intestinal parasites and vector-borne pathogens in Italian shelter and kennel dogs. Comp Immunol Microbiol Infect Dis. (2017) 51:69–75. doi: 10.1016/j.cimid.2017.04.003, PMID: 28504099

[17] Paoletti B Traversa D Iorio R De Berardinis A Bartolini R Salini R. Zoonotic parasites in feces and fur of stray and private dogs from Italy. Parasitol Res. (2015) 114:2135–41. doi: 10.1007/s00436-015-4402-6 25773179

[18] Rizk MA Abourizk N Gadhiya KP Hansrivijit P Goldman JD. A bite so bad: septic shock due to *Capnocytophaga Canimorsus* following a dog bite. Cureus. (2021) 13:e14668. doi: 10.7759/cureus.14668, PMID: 34055517 PMC8144272

[19] Mader N Lührs F Langenbeck M Herget-Rosenthal S. *Capnocytophaga canimorsus* – a potent pathogen in immunocompetent humans – systematic review and retrospective observational study of case reports. Infect Dis. (2020) 52:65–74. doi: 10.1080/23744235.2019.1687933 31709860

[20] Hannon DM Harkin E Donnachie K Sibartie S Doyle M Chan G. A case of *Capnocytophaga canimorsus* meningitis and bacteraemia. Irish J Med Sci. (2020) 189:251–2. doi: 10.1007/s11845-019-02045-0 31203505

[21] Lloret A Egberink H Addie D Belák S Boucraut-Baralon C Frymus T. *Capnocytophaga Canimorsus* infection in cats: ABCD guidelines on prevention and management. J Feline Med Surg. (2013) 15:588–90. doi: 10.1177/1098612X13489220, PMID: 23813822 PMC11148950

[22] Damborg P Broens EM Chomel BB Guenther S Pasmans F Wagenaar JA. Bacterial Zoonoses transmitted by household pets: state-of-the-art and future perspectives for targeted research and policy actions. J Comp Pathol. (2016) 155:S27–40. doi: 10.1016/j.jcpa.2015.03.004 25958184

[23] Joosten P Ceccarelli D Odent E Sarrazin S Graveland H Van Gompel L. Antimicrobial usage and resistance in companion animals: a cross-sectional study in three European countries. Antibiotics. (2020) 9:87. doi: 10.3390/antibiotics9020087 32079072 PMC7175148

[24] Adams RJ Kim SS Mollenkopf DF Mathys DA Schuenemann GM Daniels JB. Antimicrobial-resistant Enterobacteriaceae recovered from companion animal and livestock environments. Zoonoses Public Health. (2018) 65:519–27. doi: 10.1111/zph.12462 29575700

[25] Trott DJ Abraham S Adler B. Antimicrobial resistance in Leptospira, Brucella, and other rarely investigated veterinary and zoonotic pathogens. Microbiol Spectr. (2018) 6:eISSN 2165–0497. doi: 10.1128/microbiolspec.ARBA-0029-2017 PMC11633603 30027885

[26] Buhmann G Paul F Herbst W Melzer F Wolf G Hartmann K. Canine brucellosis: insights into the epidemiologic situation in Europe. Front Vet Sci. (2019) 6:151. doi: 10.3389/fvets.2019.00151 31214601 PMC6554662

[27] Lucero NE Jacob NO Ayala SM Escobar GI Tuccillo P Jacques I. Unusual clinical presentation of brucellosis caused by *Brucella canis*. J Med Microbiol. (2005) 54:505–8. doi: 10.1099/jmm.0.45928-0, PMID: 15824432

[28] Corrente M Franchini D Decaro N Greco G D’Abramo M Greco MF. Detection of *Brucella canis* in a dog in Italy. New Microbiol. (2010) 33:337–41. PMID: 21213592

[29] Mughini-Gras L Angeloni G Salata C Vonesch N D’Amico W Campagna G. Hepatitis e virus infection in North Italy: high seroprevalence in swine herds and increased risk for swine workers. Epidemiol Infect. (2017) 145:3375–84. doi: 10.1017/S0950268817002485 29145911 PMC9148764

[30] De Sabato L Di Bartolo I Lapa D Capobianchi MR Garbuglia AR. Molecular characterization of HEV genotype 3 in Italy at human/animal Interface. Front Microbiol. (2020) 11:137. doi: 10.3389/fmicb.2020.00137 32117156 PMC7014918

[31] Tei S Kitajima N Takahashi K Mishiro S. Zoonotic transmission of hepatitis E virus from deer to human beings. Lancet. (2003) 362:371–3. doi: 10.1016/S0140-6736(03)14025-1 12907011

[32] Puttini C Riccio ML Redi D Tordini G Cenerini M Romanello F. Seroprevalence of hepatitis E virus (HEV) infection in blood donors and renal transplant recipients: a retrospective study from Central Italy. Infez Med. (2015) 23:253–6. PMID: 26397295

[33] Ferri G Vergara A. Hepatitis E virus in the food of animal origin: a review. Foodborne Pathog Dis. (2021) 18:368–77. doi: 10.1089/fpd.2020.2896 33784472 PMC8215403

[34] Lhomme S Marion O Abravanel F Izopet J Kamar N. Clinical manifestations, pathogenesis and treatment of hepatitis E virus infections. J Clin Med. (2020) 9:331. doi: 10.3390/jcm9020331 31991629 PMC7073673

[35] Bernardini A Pacini MI Fonti N Forzan M Marchetti V Mazzei M. Serological, virological investigation and hepatic injury evaluation for hepatitis E virus in hunting dogs. Pathog. (2022) 11:1123. doi: 10.3390/pathogens11101123 PMC9608991 36297180

[36] Li Y Qu C Spee B Zhang R Penning LC de Man RA. Hepatitis E virus seroprevalence in pets in the Netherlands and the permissiveness of canine liver cells to the infection. Ir Vet J. (2020) 73:6. doi: 10.1186/s13620-020-00158-y 32266057 PMC7119158

[37] Bigoraj E Rzeżutka A. Hepatitis E virus in humans, farm animals and animals from the sylvatic environment. Med Weter. (2017) 73:456–61. doi: 10.21521/mw.5762

[38] Liang H Chen J Xie J Sun L Ji F He S. Hepatitis E virus serosurvey among pet dogs and cats in several developed cities in China. PLoS One. (2014) 9:98068. doi: 10.1371/journal.pone.0098068 PMC4045666 24896257

[39] Lin J Karlsson M Olofson AS Belák S Malmsten J Dalin AM. High prevalence of hepatitis e virus in Swedish moose--a phylogenetic characterization and comparison of the virus from different regions. PLoS One. (2015) 10:e0122102. doi: 10.1371/journal.pone.0122102 25906163 PMC4408071

[40] Herder V Wohlsein P Grunwald D Janssen H Meyer H Kaysser P. Poxvirus infection in a cat with presumptive human transmission. Vet Dermatol. (2011) 22:220–4. doi: 10.1111/j.1365-3164.2010.00947.x 21375609

[41] Rosone F Sala MG Cardeti G Rombolà P Cittadini M Carnio A. Sero-epidemiological survey of Orthopoxvirus in stray cats and in different domestic, wild and exotic animal species of Central Italy. Viruses. (2021) 13:2105. doi: 10.3390/v13102105 34696535 PMC8537024

[42] Lapa D Beltrame A Arzese A Carletti F Di Caro A Ippolito G. Orthopoxvirus Seroprevalence in cats and veterinary personnel in north-eastern Italy in 2011. Viruses. (2019) 11:101. doi: 10.3390/v11020101 30691058 PMC6409756

[43] Hobbs EC Reid TJ. Animals and SARS-CoV-2: species susceptibility and viral transmission in experimental and natural conditions, and the potential implications for community transmission. Transbound Emerg Dis. (2020) 68:1850–67. doi: 10.1111/tbed.13885 33091230 PMC8359434

[44] Younes S Younes N Shurrab F Nasrallah GK. Severe acute respiratory syndrome coronavirus-2 natural animal reservoirs and experimental models: systematic review. Rev Med Virol. (2020) 31:e2196. doi: 10.1002/rmv.2196 33206434 PMC7744864

[45] Hosie MJ Hofmann-Lehmann R Hartmann K Egberink H Truyen U Addie DD. Anthropogenic infection of cats during the 2020 COVID-19 pandemic. Viruses. (2021) 13:185. doi: 10.3390/v13020185 33530620 PMC7911697

[46] Sit THC Brackman CJ Ip SM Tam KWS Law PYT To EMW. Infection of dogs with SARS-CoV-2. Nature. (2020) 586:776–8. doi: 10.1038/s41586-020-2334-5 32408337 PMC7606701

[47] Natale A Mazzotta E Mason N Ceglie L Mion M Stefani A. Sars-cov-2 natural infection in a symptomatic cat: diagnostic, clinical and medical management in a one health vision. Animals. (2021) 11:1640. doi: 10.3390/ani11061640 34205893 PMC8227534

[48] Patterson EI Elia G Grassi A Giordano A Desario C Medardo M. Evidence of exposure to SARS-CoV-2 in cats and dogs from households in Italy. Nat Commun. (2020) 11:6231. doi: 10.1038/s41467-020-20097-0 33277505 PMC7718263

[49] Doliff R Martens P. Cats and SARS-CoV-2: a scoping review. Animals. (2022) 12:1413. doi: 10.3390/ani12111413 35681877 PMC9179433

[50] Salvatore M Martinez-Sobrido L Parrish C Borland S Gracieux P. Influenza a virus infection in cats and dogs: a literature review in the light of the “one health” concept. Front Public Heal. (2020) 8:83. doi: 10.3389/fpubh.2020.00083 PMC7098917 32266198

[51] Anderson TC Crawford PC Dubovi EJ Gibbs EPJ Hernandez JA. Prevalence of and exposure factors for seropositivity to H3N8 canine influenza virus in dogs with influenza-like illness in the United States. J Am Vet Med Assoc. (2013) 242:209–16. doi: 10.2460/javma.242.2.209, PMID: 23276098

[52] Luchs A Timenetsky MC. Group a rotavirus gastroenteritis: post-vaccine era, genotypes and zoonotic transmission. Einstein. (2016) 14:278–87. doi: 10.1590/S1679-45082016RB3582 27462899 PMC4943361

[53] Luchs A Cilli A Morillo SG Carmona RCC Timenetsky MCST. Rare G3P[3] rotavirus strain detected in Brazil: possible human-canine interspecies transmission. J Clin Virol. (2012) 54:89–92. doi: 10.1016/j.jcv.2012.01.025, PMID: 22398035

[54] Cavicchio L Tassoni L Zamperin G Campalto M Carrino M Leopardi S. Unexpected genetic diversity of two novel swine MRVs in Italy. Viruses. (2020) 12:574. doi: 10.3390/v12050574 32456089 PMC7290992

[55] Ghasemzadeh I Namazi SH. Review of bacterial and viral zoonotic infections transmitted by dogs. J Med Life. (2015) 8:1–5. PMID: 28316698 PMC5319273

[56] Cavicchio L Tassoni L Laconi A Cunial G Gagliazzo L Milani A. Unrevealed genetic diversity of GII norovirus in the swine population of north East Italy. Sci Rep. (2020) 10:9217. doi: 10.1038/s41598-020-66140-4 32513947 PMC7280493

[57] Overgaauw PAM Vinke CM van Hagen MAE Lipman LJA. A one health perspective on the human–companion animal relationship with emphasis on zoonotic aspects. Int J Environ Res Public Health. (2020) 17:3789. doi: 10.3390/ijerph17113789 32471058 PMC7312520

[58] Halsby KD Walsh AL Campbell C Hewitt K Morgan D. Healthy animals, healthy people: zoonosis risk from animal contact in pet shops, a systematic review of the literature. PLoS One. (2014) 9:e89309. doi: 10.1371/journal.pone.0089309, PMID: 24586679 PMC3935869

[59] Cito F Rijks J Rantsios AT Cunningham AA Baneth G Guardabassi L. Prioritization of companion animal transmissible diseases for policy intervention in Europe. J Comp Pathol. (2016) 155:S18–26. doi: 10.1016/j.jcpa.2015.01.007 25814430

[60] Pesavento PA Murphy BG. Common and emerging infectious diseases in the animal shelter. Vet Pathol. (2014) 51:478–91. doi: 10.1177/0300985813511129 24265288

[61] Thépault A Rose V Queguiner M Chemaly M Rivoal K. Dogs and cats: reservoirs for highly diverse campylobacter jejuni and a potential source of human exposure. Animals. (2020) 10:838. doi: 10.3390/ani10050838 32408633 PMC7278488

[62] Dróżdż M Małaszczuk M Paluch E Pawlak A. Zoonotic potential and prevalence of Salmonella serovars isolated from pets. Infect Ecol Epidemiol. (2021) 11:1975530. doi: 10.1080/20008686.2021.1975530, PMID: 34531964 PMC8439213

[63] Steneroden KK Hill AE Salman MD. Zoonotic disease awareness in animal shelter workers and volunteers and the effect of training. Zoonoses Public Health. (2011) 58:449–53. doi: 10.1111/j.1863-2378.2011.01389.x, PMID: 21824343

[64] Steneroden KK Hill AE Salman MD. A needs-assessment and demographic survey of infection-control and disease awareness in western US animal shelters. Prev Vet Med. (2011) 98:52–7. doi: 10.1016/j.prevetmed.2010.11.001, PMID: 21126786

[65] Fagre A Olea-Popelka F Ruch-Gallie R. Intake procedures in Colorado animal shelters. Animals. (2017) 7:38. doi: 10.3390/ani7050038 28475139 PMC5447920

[66] Thrusfield M Christley R Brown H Diggle PJ French N Howe K. Veterinary epidemiology. Fourth ed Wiley -Blackwell (2017). 1–861 p.

[67] Hoffmann B Depner K Schirrmeier H Beer M. A universal heterologous internal control system for duplex real-time RT-PCR assays used in a detection system for pestiviruses. J Virol Methods. (2006) 136:200–9. doi: 10.1016/j.jviromet.2006.05.020, PMID: 16806503

[68] Smythe LD Smith IL Smith GA Dohnt MF Symonds ML Barnett LJ. A quantitative PCR (TaqMan) assay for pathogenic Leptospira spp. BMC Infect Dis. (2002) 2:13. doi: 10.1186/1471-2334-2-13 12100734 PMC117785

[69] Boonsilp S Thaipadungpanit J Amornchai P Wuthiekanun V Bailey MS Holden MTG. A single multilocus sequence typing (MLST) scheme for seven pathogenic Leptospira species. PLoS Negl Trop Dis. (2013) 7:e1954. doi: 10.1371/journal.pntd.0001954 23359622 PMC3554523

[70] World Organization for Animal Health. Leptospirosis. Man. Diagnostic tests vaccines Terr. Anim. OIE Terr Man. (2024) 1–14:thirteenth edition 2024. Available at: https://www.woah.org/fileadmin/Home/fr/Health_standards/tahm/3.01.12_LEPTO.pdf

[71] Balboni A Mazzotta E Boniotti MB Bertasio C Bellinati L Lucchese L. Outbreak of *Leptospira borgpetersenii* serogroup Sejroe infection in kennel: the role of dogs as sentinel in specific environments. Int J Environ Res Public Health. (2022) 19:3906. doi: 10.3390/ijerph19073906 35409589 PMC8997430

[72] World Organization for Animal Health. Leishmaniosis. Man Diagnostic Tests Vaccines Terr Anim. (2021) 1–14. Chapter 3.1.11: thirteenth edition 2024. Available at: https://www.woah.org/fileadmin/Home/fr/Health_standards/tahm/3.01.11_LEISHMANIOSIS.pdf

[73] Staggemeier R Pilger DA Spilki FR Cantarelli VV. Multiplex sybr® green-real time PCR (qPCR) assay for the detection and differentiation of Bartonella henselae AND *Bartonella clarridgeiae* in cats. Rev Inst Med Trop Sao Paulo. (2014) 56:93–5. doi: 10.1590/S0036-46652014000200001 24626408 PMC4085843

[74] Pennisi MG Marsilio F Hartmann K Lloret A Addie D Belák S. Bartonella species infection in cats: ABCD guidelines on prevention and management. J Feline Med Surg. (2013) 15:563–9. doi: 10.1177/1098612X13489214, PMID: 23813816 PMC11148970

[75] Perletta F Di Pancrazio C Rodomonti D Di Febo T Luciani M Krasteva IM. Evaluation of three serological tests for diagnosis of canine brucellosis. Microorganisms. (2023) 11:2162. doi: 10.3390/microorganisms11092162 37764006 PMC10536495

[76] Friedhoff KT. Manual of veterinary parasitological laboratory techniques In: MAFF. Ministry of agriculture, fisheries and food. Her Majesty’s stationary office, vol. 4. 3rd ed. H.M. Stationery Office 1986. London, UK: Scientific Research Publishing, Inc. (1986)

[77] Taylor MA Coop RL Wall RL Otranto D Garippa G. MM. Parassitologia e malattie parassitarie degli animali. 3rd ed. EMSI, editor. Italy: EMSI (2010).

[78] Citterio CV Obber F Trevisiol K Dellamaria D Celva R Bregoli M. Echinococcus multilocularis and other cestodes in red foxes (*Vulpes vulpes*) of Northeast Italy, 2012-2018. Parasit Vectors. (2021) 14:29. doi: 10.1186/s13071-020-04520-5 33413547 PMC7789758

[79] de Hoog GS Guarro J Gene J Figueras MJ. Atlas of clinical Fungi In: Centraalbureau voor Schimmelcultures. 2nd Editio ed. Utrecht, The Netherlands: (2020)

[80] van Dam AP van Weert A Harmanus C Hovius KE Claas ECJ Reubsaet FAG. Molecular characterization of Capnocytophaga canimorsus and other canine Capnocytophaga spp. and assessment by PCR of their frequencies in dogs. J Clin Microbiol. (2009) 47:3218–25. doi: 10.1128/JCM.01246-09 19641058 PMC2756906

[81] Corman VM Landt O Kaiser M Molenkamp R Meijer A Chu DKW. Detection of 2019 novel coronavirus (2019-nCoV) by real-time RT-PCR. Eur Secur. (2020) 25:1–8. doi: 10.2807/1560-7917.ES.2020.25.3.2000045 PMC6988269 31992387

[82] Bellinati L Campalto M Mazzotta E Ceglie L Cavicchio L Mion M. One-year surveillance of SARS-CoV-2 exposure in stray cats and kennel dogs from northeastern Italy. Microorganisms. (2022) 11:110. doi: 10.3390/microorganisms11010110 36677401 PMC9866628

[83] Jothikumar N Cromeans TL Robertson BH Meng XJ Hill VR. A broadly reactive one-step real-time RT-PCR assay for rapid and sensitive detection of hepatitis E virus. J Virol Methods. (2006) 131:65–71. doi: 10.1016/j.jviromet.2005.07.004, PMID: 16125257

[84] Johne R Dremsek P Kindler E Schielke A Plenge-Bönig A Gregersen H. Rat hepatitis E virus: geographical clustering within Germany and serological detection in wild Norway rats (*Rattus norvegicus*). Infect Genet Evol. (2012) 12:947–56. doi: 10.1016/j.meegid.2012.02.021, PMID: 22554648

[85] Jiang X Huang PW Zhong WM Farkas T Cubitt DW Matson DO. Design and evaluation of a primer pair that detects both Norwalk- and Sapporo-like caliciviruses by RT-PCR. J Virol Methods. (1999) 83:145–54. doi: 10.1016/s0166-0934(99)00114-7 10598092

[86] Otto PH Rosenhain S Elschner MC Hotzel H Machnowska P Trojnar E. Detection of rotavirus species A, B and C in domestic mammalian animals with diarrhoea and genotyping of bovine species A rotavirus strains. Vet Microbiol. (2015) 179:168–76. doi: 10.1016/j.vetmic.2015.07.021 26223422

[87] Leary TP Erker JC Chalmers ML Wetzel JD Desai SM Mushahwar IK. Detection of reovirus by reverse transcription-polymerase chain reaction using primers corresponding to conserved regions of the viral L1 genome segment. J Virol Methods. (2002) 104:161–5. doi: 10.1016/S0166-0934(02)00058-7 12088825

[88] Hoffmann B Harder T Lange E Kalthoff D Reimann I Grund C. New real-time reverse transcriptase polymerase chain reactions facilitate detection and differentiation of novel A/H1N1 influenza virus in porcine and human samples. Berl Munch Tierarztl Wochenschr. (2010) 123:286–92. doi: 10.2376/0005-9366-123-286 20690540

[89] Gavrilova EV Shcherbakov DN Maksyutov RA Shchelkunov SN. Development of real-time PCR assay for specific detection of cowpox virus. J Clin Virol. (2010) 49:37–40. doi: 10.1016/j.jcv.2010.06.003 20594906 PMC9628739

[90] Day MJ Horzinek MC Schultz RD Squires RA. WSAVA guidelines for the vaccination of dogs and cats. J Small Anim Pract. (2016) 57:E1–E45. doi: 10.1111/jsap.2_12431 26780857 PMC7166872

[91] Rijks JM Cito F Cunningham AA Rantsios AT Giovannini A. Disease risk assessments involving companion animals: an overview for 15 selected pathogens taking a European perspective. J Comp Pathol. (2016) 155:S75–97. doi: 10.1016/j.jcpa.2015.08.003 26422413

[92] Stehr-Green JK Schantz PM. The impact of zoonotic diseases transmitted by pets on human health and the economy In: The veterinary clinics of North America. Small animal practice, vol. 17 (1987). 1–15. doi: 10.1016/s0195-5616(87)50601-5 3551300

[93] Amoroso MG Serra F Miletti G Cardillo L de Martinis C Marati L. A retrospective study of viral molecular Prevalences in cats in southern Italy (Campania region). Viruses. (2022) 14:2583. doi: 10.3390/v14112583 36423192 PMC9699332

[94] Morelli S Diakou A Di Cesare A Colombo M Traversa D. Canine and feline parasitology: analogies, differences, and relevance for human health. Clin Microbiol Rev. (2021) 34:e0026620. doi: 10.1128/CMR.00266-20 34378954 PMC8404700

[95] Mazzotta E De Zan G Cocchi M Boniotti MB Bertasio C Furlanello T. Feline susceptibility to leptospirosis and presence of immunosuppressive co-morbidities: first European report of *L. interrogans* serogroup Australis sequence type 24 in a cat and survey of Leptospira exposure in outdoor cats. Trop Med Infect Dis. (2023) 8:54. doi: 10.3390/tropicalmed8010054 36668961 PMC9865706

[96] Mazzotta E Bellinati L Bertasio C Boniotti MB Lucchese L Ceglie L. Synanthropic and wild animals as sentinels of zoonotic agents: a study of Leptospira genotypes circulating in northeastern Italy. Int J Environ Res Public Health. (2023) 20:1–14. doi: 10.3390/ijerph20053783 PMC10000914 36900793

[97] Sykes JE Francey T Schuller S Stoddard RA Cowgill LD Moore GE. 2023 updated ACVIM consensus statement on leptospirosis in dogs. J Vet Intern Med. (2023) 37:1966–82. doi: 10.1111/jvim.16903 37861061 PMC10658540

[98] Piredda I Sechi S Cocco R Bertoldi L Palmas B Chisu V. Isolation of *Leptospira interrogans* Serovar Canicola in a vaccinated dog without clinical symptoms. Pathogens. (2022) 11:406. doi: 10.3390/pathogens11040406 35456081 PMC9028210

[99] Gradoni L Ferroglio E Zanet S Mignone W Venco L Bongiorno G. Monitoring and detection of new endemic foci of canine leishmaniosis in northern continental Italy: an update from a study involving five regions (2018–2019). Vet Parasitol Reg Stud Rep. (2022) 27:100676. doi: 10.1016/j.vprsr.2021.100676 35012715

[100] Signorini M Cassini R Drigo M Frangipane di Regalbono A Pietrobelli M Montarsi F. Ecological niche model of Phlebotomus perniciosus, the main vector of canine leishmaniasis in North-Eastern Italy. Geospat Health. (2014) 9:193–201. doi: 10.4081/gh.2014.16 25545936

[101] Maggi RG Halls V Krämer F Lappin M Pennisi MG Peregrine AS. Vector-borne and other pathogens of potential relevance disseminated by relocated cats. Parasit Vectors. (2022) 15:415. doi: 10.1186/s13071-022-05553-8 36348395 PMC9643929

[102] Bruno F Vitale F La Russa F Reale S Späth GF Oliveri E. Retrospective analysis of Leishmaniasis in Sicily (Italy) from 2013 to 2021: one-health impact and future control strategies. Microorganisms. (2022) 10:1704. doi: 10.3390/microorganisms10091704 36144305 PMC9504907

[103] Castelli G Bruno F Caputo V Fiorella S Sammarco I Lupo T. Genetic tools discriminate strains of Leishmania infantum isolated from humans and dogs in Sicily, Italy. PLoS Negl Trop Dis. (2020) 14:e0008465. doi: 10.1371/journal.pntd.0008465 32706789 PMC7406075

[104] Panarese R Iatta R Beugnet F Otranto D. Incidence of Dirofilaria immitis and Leishmania infantum infections in sheltered dogs from Southern Italy. Transbound Emerg Dis. (2022) 69:891–4. doi: 10.1111/tbed.14025 33547868

[105] Grippi F Galluzzo P Guercio A Blanda V Santangelo F Sciortino S. Serological and molecular evidence of *Bartonella henselae* in stray cats from southern Italy. Microorganisms. (2021) 9:979. doi: 10.3390/microorganisms9050979 33946563 PMC8147212

[106] Vermeulen MJ Verbakel H Notermans DW Reimerink JHJ Peeters MF. Evaluation of sensitivity, specificity and cross-reactivity in *Bartonella henselae* serology. J Med Microbiol Microbiol Soc. (2010) 59:743–5. doi: 10.1099/jmm.0.015248-0 20223899

[107] Álvarez-Fernández A Maggi R Martín-Valls GE Baxarias M Breitschwerdt EB Solano-Gallego L. Prospective serological and molecular cross-sectional study focusing on Bartonella and other blood-borne organisms in cats from Catalonia (Spain). Parasit Vectors. (2022) 15:6. doi: 10.1186/s13071-021-05105-6 34983610 PMC8729136

[108] Neupane P Sevala S Balakrishnan N Marr H Wilson J Maggi R. Validation of *Bartonella henselae* Western immunoblotting for Serodiagnosis of Bartonelloses in dogs. J Clin Microbiol. (2020) 58:e01335–19. doi: 10.1128/JCM.01335-19 PMC7098778 31941695

[109] Koutantou M Kambas K Makka S Fournier PE Raoult D Angelakis E. Limitations of serological diagnosis of typical cat scratch disease and recommendations for the diagnostic procedure. Can J Infect Dis Med Microbiol. (2023) 2023:1–11. doi: 10.1155/2023/4222511 PMC10008113 36915870

[110] Persichetti MF Pennisi MG Vullo A Masucci M Migliazzo A Solano-Gallego L. Clinical evaluation of outdoor cats exposed to ectoparasites and associated risk for vector-borne infections in southern Italy. Parasit Vectors. (2018) 11:136. doi: 10.1186/s13071-018-2725-8 29554931 PMC5859451

[111] Sodini C Zani EM Pecora F Conte C Patianna VD Prezioso G. A case of atypical Bartonellosis in a 4-year-old immunocompetent child. Microorganisms. (2021) 9:950. doi: 10.3390/microorganisms9050950 33924906 PMC8146596

[112] Pischel L Radcliffe C Vilchez GA Charifa A Zhang XC Grant M. Bartonellosis in transplant recipients: a retrospective single center experience. World J Transplant. (2021) 11:244–53. doi: 10.5500/wjt.v11.i6.244, PMID: 34164299 PMC8218350

[113] Chomel BB Kasten RW. Bartonellosis, an increasingly recognized zoonosis. J Appl Microbiol. (2010) 109:743–50. doi: 10.1111/j.1365-2672.2010.04679.x, PMID: 20148999

[114] Chomel BB Boulouis HJ Maruyama S Breitschwerdt EB. Bartonella spp. in pets and effect on human health. Emerg Infect Dis. (2006) 12:389–94. doi: 10.3201/eid1203.050931 16704774 PMC3291446

[115] Shamekhi AF. Bartonellosis in chronic kidney disease: an unrecognized and unsuspected diagnosis. Ther Apher Dial. (2017) 21:430–40. doi: 10.1111/1744-9987.12571 28884961

[116] Picascia A Pagliuca C Sommese L Colicchio R Casamassimi A Labonia F. Seroprevalence of *Bartonella henselae* in patients awaiting heart transplant in Southern Italy. J Microbiol Immunol Infect. (2017) 50:239–44. doi: 10.1016/j.jmii.2015.05.001, PMID: 26051222

[117] Breitschwerdt EB Maggi RG Chomel BB Lappin MR. Bartonellosis: an emerging infectious disease of zoonotic importance to animals and human beings. J Vet Emerg Crit Care. (2010) 20:8–30. doi: 10.1111/j.1476-4431.2009.00496.x 20230432

[118] Biswas S Rolain JM. Bartonella infection: treatment and drug resistance. Future Microbiol. (2010) 5:1719–31. doi: 10.2217/fmb.10.133 21133691

[119] Greene CE McDermott M Jameson PH Atkins CL Marks AM. *Bartonella henselae* infection in cats: evaluation during primary infection, treatment, and rechallenge infection. J Clin Microbiol. (1996) 34:1682–5. doi: 10.1128/jcm.34.7.1682-1685.1996 8784569 PMC229094

[120] Genchi M Vismarra A Zanet S Morelli S Galuppi R Cringoli G. Prevalence and risk factors associated with cat parasites in Italy: a multicenter study. Parasit Vectors. (2021) 14:475. doi: 10.1186/s13071-021-04981-2 34526126 PMC8441231

[121] Möstl K Egberink H Addie D Frymus T Boucraut-Baralon C Truyen U. Prevention of infectious diseases in cat shelters: ABCD guidelines. J Feline Med Surg. (2013) 15:546–54. doi: 10.1177/1098612X13489210, PMID: 23813812 PMC11148948

[122] Joshi S Shallal A Zervos M. Vancomycin-resistant enterococci. Infect Dis Clin N Am. (2021) 35:953–68. doi: 10.1016/j.idc.2021.07.002 34752227

[123] Bellato A Robino P Stella MC Scarrone L Scalas D Nebbia P. Resistance to critical important antibacterials in Staphylococcus pseudintermedius strains of veterinary origin. Antibiot. (2022) 11:1758. doi: 10.3390/antibiotics11121758 PMC9774309 36551415

[124] Dalton KR Rock C Carroll KC Davis MF. One Health in hospitals: How understanding the dynamics of people, animals, and the hospital built-environment can be used to better inform interventions for antimicrobial-resistant gram-positive infections. Antimicrob Resist Infect Control. (2020) 9:78. doi: 10.1186/s13756-020-00737-2 32487220 PMC7268532

[125] Salgado-Caxito M Benavides JA Adell AD Paes AC Moreno-Switt AI. Global prevalence and molecular characterization of extended-spectrum β-lactamase producing-*Escherichia coli* in dogs and cats – A scoping review and meta-analysis. One Health. (2021) 12:100236. doi: 10.1016/j.onehlt.2021.100236 33889706 PMC8050393

[126] Van Den Bunt G Fluit AC Spaninks MP Timmerman AJ Geurts Y Kant A. Faecal carriage, risk factors, acquisition and persistence of ESBL-producing Enterobacteriaceae in dogs and cats and co-carriage with humans belonging to the same household. J Antimicrob Chemother. (2020) 75:342–50. doi: 10.1093/jac/dkz462, PMID: 31711228 PMC6966097

[127] de Sousa T Garcês A Silva A Lopes R Alegria N Hébraud M. The impact of the virulence of *Pseudomonas aeruginosa* isolated from dogs. Vet Sci. (2023) 10:343. doi: 10.3390/vetsci10050343 37235426 PMC10224390

[128] Iseppi R Di Cerbo A Messi P Sabia C. Antibiotic resistance and virulence traits in vancomycin-resistant enterococci (VRE) and extended-Spectrum β-lactamase/AmpC-producing (ESBL/AmpC) Enterobacteriaceae from humans and pets. Antibiotics. (2020) 9:152. doi: 10.3390/antibiotics9040152 32244399 PMC7235867

[129] Paul NC Moodley A Ghibaudo G Guardabassi L. Carriage of methicillin-resistant *Staphylococcus pseudintermedius* in small animal veterinarians: indirect evidence of zoonotic transmission. Zoonoses Public Health. (2011) 58:533–9. doi: 10.1111/j.1863-2378.2011.01398.x 21824350

[130] Vercelli C Della Ricca M Re M Gambino G Re G. Antibiotic stewardship for canine and feline acute urinary tract infection: an observational study in a small animal Hospital in Northwest Italy. Antibiot. (2021) 10:562. doi: 10.3390/antibiotics10050562 PMC8150826 34064943

[131] Marques C Gama LT Belas A Bergström K Beurlet S Briend-Marchal A. European multicenter study on antimicrobial resistance in bacteria isolated from companion animal urinary tract infections. BMC Vet Res. (2016) 12:213. doi: 10.1186/s12917-016-0840-3 27658466 PMC5034465

[132] Kauffman LK Petersen CA. Canine brucellosis. Vet Clin North Am Small Anim Pract. (2019) 49:763–79. doi: 10.1016/j.cvsm.2019.02.013 30961996

[133] Djokic V Freddi L de Massis F Lahti E van den Esker MH Whatmore A. The emergence of *Brucella canis* as a public health threat in Europe: what we know and what we need to learn. Emerg Microbes Infect. (2023) 12:2249126. doi: 10.1080/22221751.2023.2249126 37649455 PMC10540651

[134] Santos RL Souza TD Mol JPS Eckstein C Paíxão TA. Canine brucellosis: an update. Front Vet Sci. (2021) 8:594291. doi: 10.3389/fvets.2021.594291 33738302 PMC7962550

[135] De Massis F Sacchini F Petrini A Bellucci F Perilli M Garofolo G. Canine brucellosis due to *Brucella canis*: description of the disease and control measures. Vet Ital. (2022) 58:5–23. doi: 10.12834/VetIt.2561.16874.1 35766163

[136] Simonato G Frangipane di Regalbono A Cassini R Traversa D Beraldo P Tessarin C. Copromicroscopic and molecular investigations on intestinal parasites in kenneled dogs. Parasitol Res. (2015) 114:1963–70. doi: 10.1007/s00436-015-4385-3 25687526

[137] Chen J Liu Q Liu GH Zheng WB Hong SJ Sugiyama H. Toxocariasis: a silent threat with a progressive public health impact. Infect Dis Poverty. (2018) 7:59. doi: 10.1186/s40249-018-0437-0 29895324 PMC5998503

[138] Tamponi C Knoll S Tosciri G Salis F Dessì G Cappai MG. Environmental contamination by dog feces in touristic areas of Italy: parasitological aspects and zoonotic hazards. Am J Trop Med Hyg. (2020) 103:1143–9. doi: 10.4269/ajtmh.20-0169, PMID: 32602438 PMC7470531

[139] Márquez-Navarro A García-Bracamontes G Álvarez-Fernández BE Ávila-Caballero LP Santos-Aranda I Díaz-Chiguer DL. Trichuris vulpis (Froelich, 1789) infection in a child: a case report. Korean J Parasitol. (2012) 50:69–71. doi: 10.3347/kjp.2012.50.1.69 22451737 PMC3309054

[140] Waindok P Raue K Grilo ML Siebert U Strube C. Predators in northern Germany are reservoirs for parasites of one health concern. Parasitol Res. (2021) 120:4229–39. doi: 10.1007/s00436-021-07073-3 33547507 PMC8599236

[141] Morelli S Crisi PE Di Cesare A De Santis F Barlaam A Santoprete G. Exposure of client-owned cats to zoonotic vector-borne pathogens: clinic-pathological alterations and infection risk analysis. Comp Immunol Microbiol Infect Dis. (2019) 66:101344. doi: 10.1016/j.cimid.2019.101344 31437677

[142] Woolsey ID Miller AL. Echinococcus granulosus sensu lato and Echinococcus multilocularis: a review. Res Vet Sci. (2021) 135:517–22. doi: 10.1016/j.rvsc.2020.11.010, PMID: 33246571

[143] Tamarozzi F Legnardi M Fittipaldo A Drigo M Cassini R. Epidemiological distribution of Echinococcus granulosus s.l. infection in human and domestic animal hosts in European Mediterranean and Balkan countries: a systematic review. PLoS Negl Trop Dis. (2020) 14:e0008519. doi: 10.1371/journal.pntd.0008519 32776936 PMC7440662

[144] Frymus T Gruffydd-Jones T Pennisi MG Addie D Belák S Boucraut-Baralon C. Dermatophytosis in cats: ABCD guidelines on prevention and management. J Feline Med Surg. (2013) 15:598–604. doi: 10.1177/1098612X13489222, PMID: 23813824 PMC11148949

[145] Moretti A Agnetti F Mancianti F Nardoni S Righi C Moretta I. Dermatophytosis in animals: epidemiological, clinical and zoonotic aspects. G Ital di Dermatologia e Venereol. (2013) 148:563–72. 24442037

[146] Coombs KM. Reovirus structure and morphogenesis. Curr Top Microbiol Immunol. (2006) 309:117–67. doi: 10.1007/3-540-30773-7_5 16909899

[147] Lelli D Moreno A Lavazza A Bresaola M Canelli E Boniotti MB. Identification of mammalian Orthoreovirus type 3 in Italian bats. Zoonoses Public Health. (2013) 60:84–92. doi: 10.1111/zph.12001 22931153

[148] Arnaboldi S Righi F Filipello V Trogu T Lelli D Bianchi A. Mammalian orthoreovirus (Mrv) is widespread in wild ungulates of northern Italy. Viruses. (2021) 13:238. doi: 10.3390/v13020238, PMID: 33546342 PMC7913563

[149] Moutelíková R Prodělalová J. The possibilities of zoonotic transmission of rotaviruses. Epidemiol Mikrobiol Imunol. (2015) 64:66–71. 26099609

[150] Khamrin P Maneekarn N Peerakome S Yagyu F Okitsu S Ushijima H. Molecular characterization of a rare G3P[3] human rotavirus reassortant strain reveals evidence for multiple human-animal interspecies transmissions. J Med Virol. (2006) 78:986–94. doi: 10.1002/jmv.20651, PMID: 16721863

[151] German AC Iturriza-Gómara M Dove W Sandrasegaram M Nakagomi T Nakagomi O. Molecular epidemiology of rotavirus in cats in the United Kingdom. J Clin Microbiol. (2015) 53:455–64. doi: 10.1128/JCM.02266-14 25411173 PMC4298538

[152] Doro R Farkas SL Martella V Banyai K. Zoonotic transmission of rotavirus: surveillance and control. Expert Rev Anti Infect Ther. (2015) 13:1337–50. doi: 10.1586/14787210.2015.1089171 26428261

[153] Bialasiewicz S Duarte TPS Nguyen SH Sukumaran V Stewart A Appleton S. Rapid diagnosis of *Capnocytophaga canimorsus* septic shock in an immunocompetent individual using real-time Nanopore sequencing: a case report. BMC Infect Dis. (2019) 19:660. doi: 10.1186/s12879-019-4173-2 31340776 PMC6657077

[154] Mendes FR Bruniera FR Schmidt J Cury AP Rizeck C Higashino H. *Capnocytophaga sputigena* bloodstream infection in hematopoietic stem cell transplantations: two cases report and review of the literature. Rev Inst Med Trop Sao Paulo. (2020) 62:1–4. doi: 10.1590/s1678-9946202062048 PMC7359719 32667390

[155] McNamara T Richt JA Glickman L. A critical needs assessment for research in companion animals and livestock following the pandemic of COVID-19 in humans. Vector Borne Zoonotic Dis. (2020) 20:393–405. doi: 10.1089/vbz.2020.2650 32374208 PMC7249469

[156] Filipe J Lauzi S Marinoni V Servida F Dall’Ara P. Zoonoses and pet owners: a survey on risk perception in Northern Italy. Comp Immunol Microbiol Infect Dis. (2024) 112:102224. doi: 10.1016/j.cimid.2024.102224 39053041

[157] McCobb EC Patronek GJ Marder A Dinnage JD Stone MS. Assessment of stress levels among cats in four animal shelters. J Am Vet Med Assoc. (2005) 226:548–55. doi: 10.2460/javma.2005.226.548, PMID: 15742695

[158] Squires RA Crawford C Marcondes M Whitley N. 2024 guidelines for the vaccination of dogs and cats - compiled by the Vaccination Guidelines Group (VGG) of the World Small Animal Veterinary Association (WSAVA). J Small Anim Pract. (2024) 65:277–316. doi: 10.1111/jsap.13718, PMID: 38568777

