# Supplementary material for: Zoonoses in dog and cat shelters in North-East Italy: update on emerging, neglected and known zoonotic agents
Source: Front Vet Sci. 2024 Nov 27;11:1490649. doi: 10.3389/fvets.2024.1490649 (PMC11631924; doi:10.3389/fvets.2024.1490649)
Supplement: Supplementary file 1 [file Table_1.DOCX]

**Table A**: List of references, molecular methods, primers and probes, and thermal profiles used to detect each pathogen included in the research project:

| **Pathogens with target gene and references** | **Molecular method used** | **Sequences and identification of primers and probes** | **Thermal Profile / Positivity cut-off for Real time PCR** |
| --- | --- | --- | --- |
| COWPOX VIRUS  ORF D11L (128bp)  Modified by Gavrilova et al., 2010  DOI: [10.1016/j.jcv.2010.06.003](https://doi.org/10.1016/j.jcv.2010.06.003) | Real time PCR with TaqMan probe | CPX FOR  5’-AAAACTCTCCACTTTCCATCTTCT-3’ | Activation at 95° C for 5’  Denaturation at 95° C for 15’’  Annealing at 65° C for 30’’  Repeat for 40 cycles |
|  |  | CPX REV  5’-GCATTCAGATACGGATACTGATTC-3’ | Cut-off  Ct. ≤ 35 |
|  |  | CPX PROBE  5’- FAM-CCACAATCAGGATCTGTAAAGCGAGC-3’-BHQ1 |  |
| INFLUENZA VIRUS type A  Gene M (99bp)  Hoffmann et al., 2010  DOI: [10.2376/0005-9366-123-286](https://doi.org/10.2376/0005-9366-123-286) | Real time RT-PCR with TaqMan probe | IAV M1-F  5’-AGATGAGTCTTCTAACCGAGGTCG-3’ | Reverse Transcription 45° C for 10’  Activation/inactivation at 95° C for 10’  Denaturation at 95° C for 15’’  Annealing/ extension at 55° C for 20’’  Repeat for 42 cycles |
|  |  | IAV-M1.1 R  5’-TGC AAA AAC ACT TTC AAG TYT CTG-3’ |  |
|  |  | IAV-M1.2-R  5’- TGC AAA GAC ACT TTC CAG TCT CTG-3’ | Cut-off  Ct. ≤ 35 |
|  |  | Probe IAV – M1-FAM  5’- FAM -TCAGGCCCCCTCAAAGCCGA- 3’ BHQ1 |  |
| MAMMALIAN ORTHOREOVIRUS  L1 gene (416bp)  Modified Leary et al., 2002  DOI: [10.1016/s0166-0934(02)00058-7](https://doi.org/10.1016/s0166-0934(02)00058-7) | End - point RT PCR | L1-rv5F  5’-GCATCCATTGTAAATGACGAGTCTG-3’ | Reverse transcription at 50°C for 30’  Activation at 95° C for 15’  Denaturation at 94° C for 30’’  Annealing at 52° C for 30’’  Extension at 72°C for 40’’  Repeat for 44 cycles  Final elongation at 72°C for 5’ |
|  |  | L1-rv6R  5’-CTTGAGATTAGCTCTAGCATCTTCTG-3’ |  |
| NOROVIRUS  RdRP (300bp)  Jiang et al.,1999  DOI: [10.1016/s0166-0934(99)00114-7](https://doi.org/10.1016/s0166-0934(99)00114-7) | End - point RT PCR and Sanger Sequencing | p290  5’-GATTACTCCAAGTGGGACTCCAC-3’ | Reverse transcription at 55°C for 30 min  Denaturation at 94°C for 2’  Activation at 94°C for 30’’  Annealing at 48°C for 30’’  Extension at 68°C for 20’’  Final elongation at 68°C for 5’  Repeat for 35 cycles |
|  |  | p110  5’-ACDATYTCATCATCACCATA-3’ |  |
| ROTAVIRUS TIPO A  Nsp3 (78bp)  Pang et al., 2004  DOI: 10.1002/jmv.20009 | Real time RT-PCR with TaqMan probe | RVA7-1F  5’-RCATRACCCYCTATGAGCAC-3’ | Reverse Transcription 50° C for 20’  Activation/inactivation at 95° C for 15’  Denaturation at 94° C for 45’’  Annealing/ extension at 60° C for 45’’  Repeat for 45 cycles |
|  |  | Rota NVP3-R  5’- GGTCACATAACGCCCC-3’ | Cut-off  Ct. ≤ 38 |
| Otto et al., 2015  DOI: [10.1016/j.vetmic.2015.07.021](https://doi.org/10.1016/j.vetmic.2015.07.021) |  | RVA7 probe  5’- FAM -ATAGTTAAAAGCTAACACTGTCAAAAACCTAAA-3’BHQ1 |  |
| JHEV (Orthohepevirus A)  ORF 3 (70bp)  Jothikumar et al. 2006 https://doi.org/10.1016/j.jviromet.2005.07.004 | Real time RT-PCR with TaqMan probe | JHEV F  5'- GGT GGT TTC TGG GGT GAC -3' | Reverse Transcription 45° C for 10’  Activation/inactivation at 95° C for 10’  Denaturation at 95° C for 15’’  Annealing at 56° C for 20’’  Extension at 72° C for 30’’  Repeat for 42 cycles |
|  |  | JHEV PROBE  5' -6FAM - TGA TTC TCA GCC CTT CGC 3' - BHQ1 | Cut-off  Ct. ≤ 38 |
|  |  | JHEVR  5'- AGG GGT TGG TTG GAT GAA -3' |  |
| RHEV (Orthohepevirus C)  73bp  Johne et al. 2012  https://doi.org/10.1016/j.meegid.2012.02.021 | Real time RT-PCR with TaqMan probe | rHEV-F  5' - TAC CCG ATG CCG GGC AGT - 3' | Reverse Transcription 45° C for 10’  Activation/inactivation at 95° C for 10’  Denaturation at 95° C for 15’’  Annealing at 56° C for 20’’  Extension at 72° C for 30’’  Repeat for 42 cycles |
|  |  | rHEV-R2  5' - ATC YAC ATC WGG GAC AGG - 3' | Cut-off  Ct. ≤ 38 |
|  |  | rHEV-P2  5' 6FAM - AAT GAC AGC ACA GGC ACC GGC GCC 3' - BHQ1 |  |
| LEPTOSPIRA Spp.  rrs 16S gene (87bp)  Smythe et al. 2002  https://doi.org/10.1186/1471-2334-2-13 | Real time PCR with TaqMan probe | LEPTO F  5’ CCC GCG TCC GAT TAG 3’ | Activation at 95° C for 10’  Denaturation at 95° C for 15’’  Annealing/ extension at 60° C for 60’’  Repeat for 40 cycles |
|  |  | LEPTO R  5' TCC ATT GTG GCC GRA CAC 3’ | Cut-off  Ct. ≤ 38 |
|  |  | LEPTO PROBE  5’FAM- CTC ACC AAG GCG ACG ATC GGT AGC -TMR 3’ |  |
| SARS-COV-2  V.M. Corman, et al. Euro Surveill. 2020 Jan;25(3):2000045  https://doi.org/10.2807/1560-7917.es.2020.25.3.2000045 | Real time RT-PCR with TaqMan probe | E_Sarbeco_F  5’-ACAGGTACGTTAATAGTTAATAGCGT- 3’ | Reverse Transcription 50° C for 30’  Activation/inactivation at 95° C for 10’  Denaturation at 95° C for 15’’  Annealing at 60° C for 60’’  Repeat for 45 cycles |
|  |  | E_Sarbeco_R  5’-ATATTGCAGCAGTACGCACACA- 3’ |  |
|  |  | E_Sarbeco_P1  5’-6FAM-ACACTAGCCATCCTTACTGCGCTTCG-BHQ1- 3’ |  |
|  |  | N_Sarbeco_F  5’-CACATTGGCACCCGCAATC-3’ |  |
|  |  | N_Sarbeco_R  5’-GAGGAACGAGAAGAGGCTTG-3’ |  |
|  |  | N_Sarbeco_P1  FAM-5’-ACTTCCTCAAGGAACAACATTGCCA-BBQ-3’ | Cut-off  Ct. ≤ 40 |
|  |  | RdRP_SARSr-F  5’-GTGARATGGTCATGTGTGGCGG-3’ |  |
|  |  | RdRP_SARSr-R  5’-CARATGTTAAASACACTATTAGCATA-3’ |  |
|  |  | RdRP_SARSr-P1  FAM-5’CCAGGTGGWACRTCATCMGGTGATGC-BBQ-3’ |  |
|  |  | RdRP_SARSr-P2  FAM-5’-CCAGGTGGAACCTCATCAGGAGATGC-BBQ-3’ |  |
| Universal positive internal control EGFP  Hoffmann, B 2006  https://doi.org/10.1016/j.jviromet.2006.05.020 | Real time RT PCR, TaqMan Probe | EGFP 1-F  5 ’- GAC CAC TAC CAG CAG AAC AC - 3’ | Reverse Transcription 45° C for 10’  Activation/inactivation at 95° C for 10’  Denaturation at 95° C for 15’’  Annealing/ extension at 55° C for 20’’  Extension at 72°C for 30’’  Repeat for 42 cycles |
|  |  | EGFP 2-R  5’- GAA CTC CAG CAG GAC CAT G - 3’ | Cut-off  Ct. ≤ 36 |
|  |  | EGFP-Probe  HEX 5’- AGC ACC CAG TCC GCC CTG AGC A - BHQ1 3’ |  |
| *Capnocytophaga canimorsus*  *rpoB* gene  Van Dam et al. 2009  DOI: [10.1128/JCM.01246-09](https://dx.doi.org/10.1128%2FJCM.01246-09) | Real time PCR TaqMan Probe | C. Cani FOR  5’-TTTCAGCTTCATTAATTCCTTTCC-3’ | Activation at 95° C for 5’  Denaturation at 95° C for 15’’  Annealing/ extension at 63° C for 30’’  Repeat for 49 cycles |
|  |  | C. Cani REV  5’-GCCTGACGCATCATATTCG-3’ |  |
|  |  | C. Cani Probe  5’-FAM-CGATGATGCGAACCGTGCGTTGAC-TAM-3’ |  |
| *Capnocytophaga cynodegmi*  *rpoB* gene  Van Dam et al. 2009  DOI: [10.1128/JCM.01246-09](https://dx.doi.org/10.1128%2FJCM.01246-09) | Real time PCR TaqMan Probe | C. Cyno FOR  5’-GAATTTCGGCTTCATTGATTCC-3’ | Cut-off  Ct. ≤ 35 |
|  |  | C. Cyno REV  5’-CGCATCATATTTGACCCCATC-3’ |  |
|  |  | C. Cyno Probe  5’-FAM-CTTGGAACACGATGATGCGAACCG-TAM-3’ |  |
